# Genome-Wide Screens for *In Vivo* Tinman Binding Sites Identify Cardiac Enhancers with Diverse Functional Architectures

**DOI:** 10.1371/journal.pgen.1003195

**Published:** 2013-01-10

**Authors:** Hong Jin, Robert Stojnic, Boris Adryan, Anil Ozdemir, Angelike Stathopoulos, Manfred Frasch

**Affiliations:** 1 University of Erlangen-Nuremberg, Department of Biology, Division of Developmental Biology, Erlangen, Germany; 2 Cambridge Systems Biology Centre and Department of Genetics, University of Cambridge, Cambridge, United Kingdom; 3 Division of Biology, California Institute of Technology, Pasadena, California, United States of America; Stowers Institute for Medical Research, United States of America

## Abstract

The NK homeodomain factor Tinman is a crucial regulator of early mesoderm patterning and, together with the GATA factor Pannier and the Dorsocross T-box factors, serves as one of the key cardiogenic factors during specification and differentiation of heart cells. Although the basic framework of regulatory interactions driving heart development has been worked out, only about a dozen genes involved in heart development have been designated as direct Tinman target genes to date, and detailed information about the functional architectures of their cardiac enhancers is lacking. We have used immunoprecipitation of chromatin (ChIP) from embryos at two different stages of early cardiogenesis to obtain a global overview of the sequences bound by Tinman *in vivo* and their linked genes. Our data from the analysis of ∼50 sequences with high Tinman occupancy show that the majority of such sequences act as enhancers in various mesodermal tissues in which Tinman is active. All of the dorsal mesodermal and cardiac enhancers, but not some of the others, require *tinman* function. The cardiac enhancers feature diverse arrangements of binding motifs for Tinman, Pannier, and Dorsocross. By employing these cardiac and non-cardiac enhancers in machine learning approaches, we identify a novel motif, termed CEE, as a classifier for cardiac enhancers. *In vivo* assays for the requirement of the binding motifs of Tinman, Pannier, and Dorsocross, as well as the CEE motifs in a set of cardiac enhancers, show that the Tinman sites are essential in all but one of the tested enhancers; although on occasion they can be functionally redundant with Dorsocross sites. The enhancers differ widely with respect to their requirement for Pannier, Dorsocross, and CEE sites, which we ascribe to their different position in the regulatory circuitry, their distinct temporal and spatial activities during cardiogenesis, and functional redundancies among different factor binding sites.

## Author Summary

The *Drosophila* homeodomain protein Tinman was the first transcription factor found to control the development and differentiation of the heart in any species. In spite of that, our knowledge of the number, identities, and mode of regulation of the downstream target genes of Tinman that are necessary to exert its cardiogenic functions is still very incomplete. To address these issues, we have performed a genome-wide analysis of DNA regions associated with Tinman-binding in embryos and the genes linked to them. The combined data from our in-depth *in vivo* assays of sequence elements with high Tinman occupancy allow the following general conclusions: (1) The majority of such sequences are active as regulatory elements (called enhancers) in mesodermal tissues that include Tinman-expressing cells. (2) The enhancers active in the heart progenitor cells and the heart generally are dependent on *tinman* gene activity, whereas those active in non-cardiac mesoderm are often bound neutrally by Tinman. (3) Tinman binding motifs in most cases are essential for cardiac enhancer activity, but in some cases they can be functionally-redundant with those of other cardiogenic factors. (4) Tinman-occupied cardiac enhancers are enriched for a newly discovered binding motif for an unknown factor that is functional *in vivo*.

## Introduction

Early cardiogenesis in *Drosophila* relies on a regulatory network of evolutionarily conserved transcription factors and closely integrated signalling events. The key cardiogenic transcription factors include the NK homeodomain factor Tinman (Tin), the GATA factor Pannier (Pnr), and the Dorsocross T-box factors (Doc1, -2 and -3). Loss of function of either of these factors results in a complete failure of heart formation (reviewed in [1], [2]). In vertebrates, closely related factors such as the NK homeodomain factor Nkx2-5, GATA4, -5, and -6, and Tbx2, -3, and -5 are likewise known to play prominent roles during early development of the normal heart and in congenital heart disease (reviewed in [3]). In *Drosophila*, *tinman* is active as the earliest among these three types of cardiogenic genes as it is initially activated during gastrulation by the bHLH factor Twist in almost the entire mesoderm [4], [5]. After gastrulation and mesoderm spreading, Tin protein is then required in conjunction with Dpp signals from the dorsal ectoderm for inducing the expression of *pnr* and maintaining the expression of its own gene, *tin*, in the dorsal mesoderm [6], [7]. During the same time, the expression of the *Doc* genes is induced by combinatorial Dpp and Wg signals, independently of *tin* and *pnr*, in segmentally-repeated areas within the dorsal mesoderm [8]. Within these areas, the three cardiogenic genes then maintain each others' expression via cross-regulatory interactions in the presence of perduring Dpp signals. In these cells of the cardiogenic mesoderm, each of the three classes of cardiogenic factors is critically required for the induction of cardiomyocyte progenitors (also known as cardioblasts). In the developing heart, the expression of *tin* and *Doc*, but not *pnr*, is maintained in cardioblasts and, in the case of *tin*, also in a subset of non-contractile pericardial cells. Unlike during the earlier stages, *tin* and *Doc* are expressed in mutually-exclusive subsets of cardioblasts within the heart, which involves cross-repressive interactions [9]. *tin* is expressed in four out of a total of six bilateral pairs of cardioblasts of the heart in each segment and is needed for specifying them as “working” cardiomyocytes, whereas *Doc* is expressed in the remaining two pairs and regulates their differentiation into inflow valves (ostia).

In accordance with its early and more widespread expression, first in almost the entire mesoderm and then in the whole dorsal mesoderm, *tin* fulfills functions in addition to cardiogenesis during mesoderm patterning and mesodermal tissue development (reviewed in [10]). In the dorsal mesoderm, *tin* is required for the formation of all somatic muscle founder cells and their corresponding muscles. Moreover, in the dorsal segmental areas between the segmental cardiac primordia *tin* is essential for the formation of the primordia of the trunk visceral mesoderm. In ventral-lateral regions of the somatic mesoderm *tin* is also required for the formation of muscle founders, albeit only for a specific subset of them. Lastly, in mesodermal areas along the ventral midline, *tin* is required for the formation of glia-like cells, termed Dorsal Median cells. Importantly, during all these events including cardiogenesis, *tin* has an obligatory requirement for additional, spatially-restricted factors and signalling effectors to fulfill its functions. This requirement is most evident from the observations that, in the normal situation, defined responses to *tin* only occur in specific subareas within Tin-expressing domains, and that forced uniform expression of *tin* in the mesoderm produces only minor alterations during mesodermal tissue development [11]. The molecular basis for these combinatorial and synergistic requirements has been clarified most extensively for the activation of the homeobox genes *even-skipped* (*eve*) in specific dorsal muscle founders as well as pericardial progenitors and *bagpipe* (*bap*) in the trunk visceral mesoderm primordia. Both *eve* and *bap* are direct targets of *tin* that contain essential Tin binding sites within their respective mesodermal enhancers. In addition, however, the *eve* enhancer (MHE/EME) requires sites for the pan-mesodermal regulator Twist, Smad binding sites targeted by Dpp, TCF/Pan sites that mediate both derepression and activation in cells receiving Wg signals, and Ets domain binding motifs likely mediating inputs from receptor tyrosine kinases [12]–[14]. Likewise, the *bap* enhancer additionally requires Smad sites for its activation (in cells receiving Dpp but not Wg signals) and sites for the forkhead domain repressor Sloppy paired (Slp) for blocking its activation by Tin and Smads (in cells receiving both Dpp and Wg signals) [15].

A number of additional direct *tin* target genes have been defined by using candidate approaches based upon genetic and expression data, particularly with respect to heart development. These include genes encoding various cardiac transcription factors (*tin*, *pnr*, *Hand*, *svp*, *mef2*, *mid*) [6], [7], [16]–[19], an ion channel subunit (*Sur*) [20], [21], a cytoskeletal component (*b3-tubulin*) [22], and a transmembrane receptor (*Toll*) [23]. All these genes fulfill stringent criteria for cardiac Tin target genes, i.e., loss (or strong reduction) of gene expression in *tin* mutant backgrounds, Tin binding to a linked cardiac enhancer element, and loss (or strong reduction) of cardiac enhancer activity upon mutation of the Tin binding motifs. In most of these cases, only the role of Tin has been defined at the level of the respective enhancers, but their detailed functional architectures have not been examined. However, in a few cases additional direct and obligatory co-regulators were described, in particular Smads (for the tinD enhancer of *tin*) [7], Pnr (for a heart enhancer of *Hand*) [16], and Doc (for a heart enhancer of *Toll*) [23].

Although the candidate approach-based studies were successful in providing a basic framework of the regulatory circuits during heart development downstream of *tin*, for a more complete picture of the roles of *tin* it is necessary to recover functionally important *tin* target genes at a more global level. A profitable starting point towards this goal can be the genome-wide identification of the DNA sequences to which Tin is bound *in vivo*. In our present study, we have taken this directed approach by performing chromatin immunoprecipitations (ChIPs) of Tin-bound sequences from embryos during early cardiogenesis, analyzing them globally, and dissecting selected examples of them functionally. Similar approaches have been initiated by others in parallel to ours [24], [25], [26], but overall, information about the functionality of Tin binding to distinct sequences is still rather limited.

Herein, we present an analysis of the Tin-bound regions and their linked genes in embryos during mesoderm patterning (3–5.5 h after egg laying) and during specification of heart, visceral and somatic muscle progenitors (5–8 h AEL). After their global characterization, which shows that the linked genes are associated preferentially with expression and functions in various mesodermal tissues and, unexpectedly, also in ectodermal tissues, we present data from our functional *in vivo* analysis of ∼50 Tin-bound sequences. The overwhelming majority of these sequences, which were selected largely among the ∼250 regions with highest levels of Tin occupancy (with the omission of sequences linked to known genes with ectodermal activities), showed enhancer activities in various mesodermal tissues, including the entire early mesoderm, dorsal and cardiac mesoderm, heart, visceral mesoderm, somatic mesoderm, and Dorsal Median cells. To test whether Tin binding is functionally relevant, we focused on the subset of enhancers active in the dorsal and cardiac mesoderm and/or the heart and demonstrate that all enhancers of this class require functional *tin* for being active. For a representative subset, we tested the *in vivo* requirements for their Tin binding motifs, as well as the requirements for the Pnr and Doc binding motifs that were also present in these enhancers. Further, by using machine learning approaches we identified a novel motif as a classifier of cardiac enhancers and show that in three out of eight tested enhancers this motif is essential for their cardiac activity. Altogether, our data indicate that high-level Tin binding signifies mesodermal enhancer activity, and at least with respect to highly-occupied cardiac enhancers, *in vivo* binding of Tin is almost always essential, although it can be functionally-redundant with other bound cardiogenic factors. We discuss the large architectural diversities of the Tin-bound cardiac enhancers in terms of the arrangement and requirement of the binding motifs for the various cardiogenic factors as well as more general aspects regarding the interpretation of genome-wide binding data from ChIP analyses.

## Results

### Identification of genome-wide *in vivo* targets of Tin

**Figure 1:**
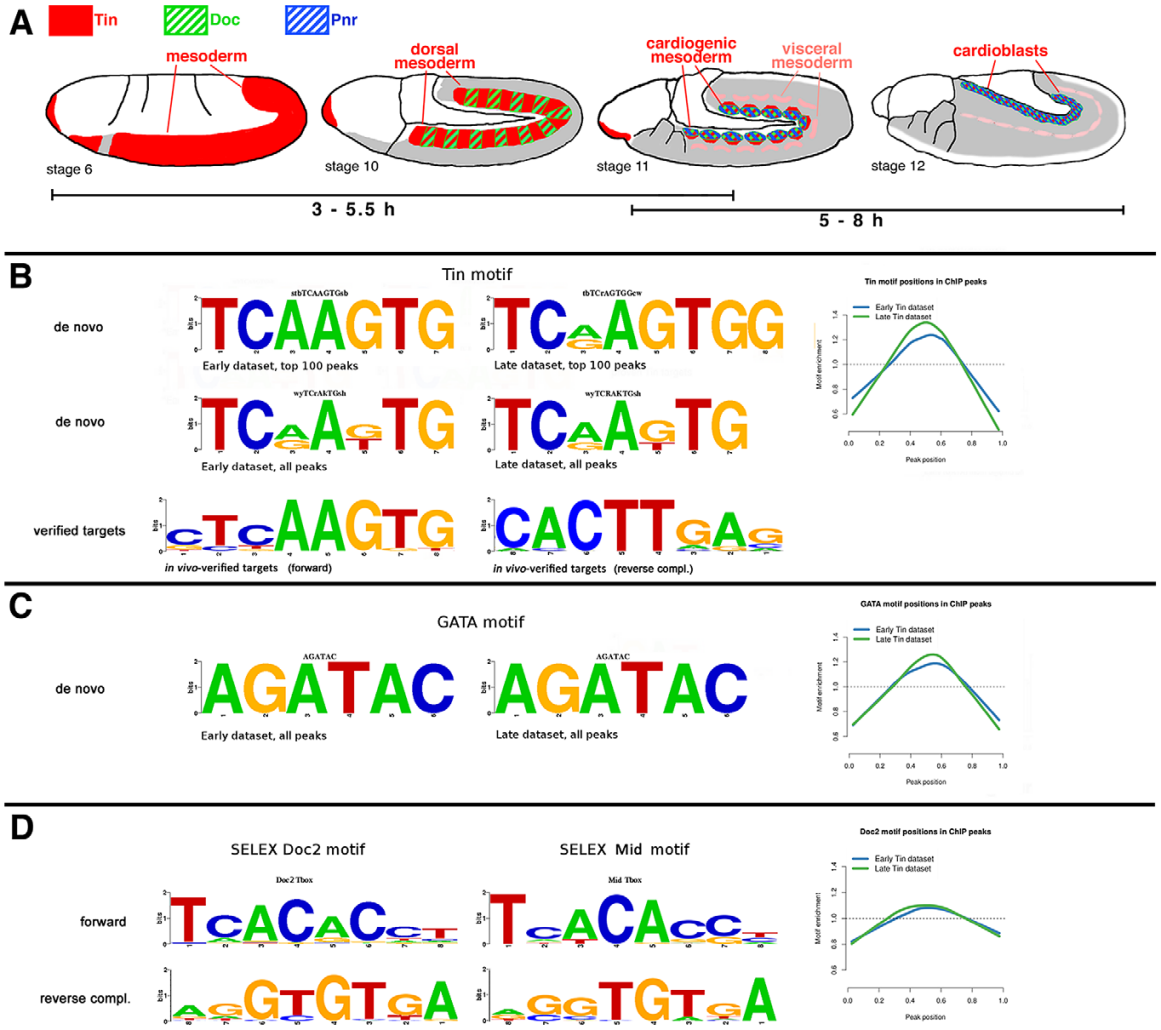
Sequence motifs enriched in Tin binding regions. (A) Schematic drawings of the expression domains of Tinman (red), Doc (green, hatched), and Pnr (blue, hatched) during the embryonic stages used for chromatin preparations. Grey: mesoderm not expressing any of these factors. (B) Tin motif recovered using *de novo* motif discovery in the top 100 peaks and full Early and Late datasets. The Tin motifs discovered in the full Early and Late dataset are almost identical, while the ones from top 100 peaks show some differences. These Tin motifs closely resemble the motifs derived from published Tin binding sites with verified *in vivo* functions (bottom) and previously published Tinman/Nkx2-5 motifs [80]. The Tin motif from the full datasets is preferentially located in the center (0.5 on X-axis) of ChIP peaks (see Materials and Methods). (C) The AGATAC motif is the most enriched sixmer in both Early and Late datasets. The core of this motif is the GATA sequence to which a number of TFs, including GATA factor Pnr, bind to. The AGATAC motif is preferentially located in the centre of ChIP peaks similarly to the Tin motif, but to a slightly lesser extent. (D) The binding motifs of the T-box factors Mid and Doc2 from SELEX experiments. The Doc2 motif is also located preferentially near the ChIP peak centre, but to a lesser extent than both Tin and GATA motifs.

Genome-wide binding sites for Tin in embryos were determined for two developmental windows, 3–5.5 h and 5–8 h after egg laying. During the 3–5.5 h window (herein termed “Early”), Tin is expressed initially in the entire mesoderm (except for the hematopoetic mesoderm) and then becomes restricted to bilateral dorsal mesodermal areas under the influence of Dpp signals (Figure 1A). During this stage, Tin is known to fulfill key roles in the early specification of heart progenitors (both cardioblasts and pericardial cells), the trunk visceral mesoderm, all dorsal somatic muscle founder cells and specific ventrolateral founders, as well as ventrally-located glia-like mesodermal cells, termed Dorsal Median (DM) cells. During the 5–8 h window (termed “Late”), the expression of Tin narrows from the broad dorsal mesodermal domain to the cardiogenic mesoderm and developing cardioblasts along the dorsal margin of the mesoderm, as well as to segmental subsets of trunk visceral mesoderm cells (Figure 1A). At this stage, Tin continues to act in the determination and diversification of cardiac cells (reviewed in [1]). The only non-mesodermal expression of Tin, which is present during both time windows, occurs in the ectodermal anlagen of the foregut (Figure 1A).

Chromatin immunoprecipitations were performed with our high quality, immunopurified Tin antibody [4], assayed by microarray hybridizations (“ChIP-chip”), and binding peaks were called using MAT (see Materials and Methods). The 3–5.5 h dataset included 2536 genome-wide binding peaks. The 5–8 h dataset yielded 983 binding peaks, of which 846 overlapped with peaks from the 3–5.5 h dataset. The lower number of peaks in the “Late” dataset above threshold is possibly due to the smaller proportion of Tin-expressing cells in embryos of this time window, which results in lower signal-to-noise ratios. In both datasets, almost half of the Tin-occupied regions were found in introns, ∼40% in intergenic regions, and most of the remaining ones in 5′UTR-encoding portions of genes (Figure S1A, S1B; Table S1). 16% of the peak summits were found in promoter regions (0 kb to −2 kb) in both datasets. If the binding regions are assigned to the nearest gene (in either direction) or exons (for intronic peaks), there are 1530 putative Tin target genes in the 3–5.5 h dataset and 679 in the 5–8 h dataset, of which 613 are shared between both sets (Table S1).

We compared our binding regions with those obtained in an independent study of Tin in comparable developmental windows (Zinzen et al. 2009, [25]). About 34% of the binding regions in our 3–5.5 h dataset overlapped with those in the 4–6 h dataset from Zinzen et al., and ∼60% of our binding regions in the 5–8 h dataset overlapped with those in their 6–8 h dataset. Conversely, ∼92% of their 4–6 h binding regions and ∼63% of their 6–8 h binding regions overlapped with those from our “Early” and “Late” datasets, respectively (Figure S1C, S1D).

We also compared our Tin-bound regions with highly occupied target (HOT) regions, which are regions displaying a binding complexity of >8 transcription factors of diverse functions during blastoderm stages, as determined by the ModENCODE Consortium [27]. Notably, a significant portion of binding regions from both of our datasets, namely ∼38%, overlapped with HOT regions (Figure S1D).

To obtain a picture of the type of genes that are bound by Tin preferentially we searched for over-represented Gene Ontology (GO) terms. As shown in Table S2A, genes encoding transcription factors and developmental genes were strongly enriched among the Tin-bound genes, both in the 3–5.5 h and the 5–8 h datasets. Of note, the GO terms “heart development”, “mesoderm development” and “gastrulation” that are particularly relevant to the known functions of *tin* ranked among the top 25 in both lists. An analysis for encoded protein domains that are enriched among the Tin-bound genes showed a preponderance of various types of DNA-binding domains at the top of both datasets. In addition, genes likely encoding components of signalling cascades such as protein kinases, immunoglobulin domains, EGF-like regions, and leucine-rich domains ranked near the top of the lists (Table S2B).

We also addressed the question of whether the Tin-bound genes were expressed preferentially in the tissues in which *tin* is expressed and is known to exert its genetic functions. For this purpose, we used the temporal and spatial annotations from the 3833 genes in the BDGP embryonic *in situ* hybridization database that had yielded “acceptable quality” expression data [28]. Indeed, there was a significant enrichment of the terms “trunk mesoderm primordium”, “visceral mesoderm primordium”, and “cardiac mesoderm primordium” among the Tin-bound genes both from the “Early” and “Late” datasets. In addition, terms reflecting the ectodermal and mesodermal expression domains of Tin in the head region (ectodermal foregut primordia and head mesoderm) were highly enriched (Table S3A). Unexpectedly, when all expression terms were considered, terms related to ectodermal and neuronal expression domains turned out to be among the most highly enriched in both of our datasets (Table S3B). This was not only the case for ectodermal domains of the head, which could overlap with Tin expression, but also for ectodermal domains of the trunk including the ventral nerve cord that exclude Tin expression. As this enrichment also holds true for the subset of genes containing intronic Tin-binding regions it is unlikely to be a result of incorrect assignments of flanking genes to the obtained binding peaks. It is also unlikely that this enrichment is an artefact due to biases in gene lengths [29], as we do not detect any major length differences between mesodermally and ectodermally/neuronally expressed genes, and terms encompassing genes with much longer average lengths (e.g., “tracheal primordium”), were not enriched (data not shown). Another possible explanation of these results could be that expression within Tin^+^ tissues is linked preferentially to expression in ectodermal and neuronal tissues, but upon omission of the genes with Tin-overlapping expression domains there is still a strong bias for ectodermal and neuronal expression. Likewise, the omission of the genes associated with Tin binding in HOT regions, which may potentially reflect non-functional (neutral) binding [50] and could have produced an artificial bias, did not change the enrichment for ectodermal tissues (data not shown). It is possible that the observed enrichment for Tin-bound genes with ectodermal domains reflects a role for Tin in repressing ectodermal genes but currently, genetic evidence for such a role is lacking. Alternatively, for unknown reasons non-functional binding of Tin could occur preferentially in ectodermally-expressed genes. One hypothesis is that Tin can occupy potential binding sites for the NK-2 type transcription factor Ventral nervous system defective (Vnd), with which it shares a very similar binding motif [30] and which may be bound by Tin if the chromatin is not masked in mesodermal tissues. Interestingly, the majority of Tin peaks in both early and late conditions are localized in open chromatin when compared to the genome-wide DNase I footprint profile generated by the BDTNP for whole embryos [31], that is, 91% of Tin Early peaks overlap DNA accessibility sites at stage 9 and 93% of Tin Late peaks overlap DNA accessibility sites at stage 11.

### Tin and GATA factor binding motifs are enriched in Tin-bound regions

To determine whether the Tin-bound regions contained any prevalent sequence motifs we performed a *de novo* motif search using Regulatory Sequence Analysis Tools (RSAT) [32] on repeat-masked DNA [33] from 3–5.5 h and 5–8 h Tin peak regions. With both the top 100 peaks and the total datasets from each developmental window, we retrieved the known Tin binding motif, which closely resembled the motif obtained with functionally-tested Tin target sites that were published at the beginning of our study (Figure 1B; Table S5). In addition, the consistently most enriched six-mer core motif AGATAC closely resembles the motif bound by various GATA factors, including that of the vertebrate cardiogenic factor GATA-6 (Figure 1C). In *Drosophila*, the GATA-4/-5/-6-related factor Pannier (Pnr) is known to play a key role in early heart development [34]–[36]. We found that the *de novo* motifs presumed to bind Tin and GATA (Pnr) are located preferentially in the center of enriched binding regions (Figure 1B and 1C). This preference was less pronounced for the motifs of the cardiac T-box factor Dorsocross 2 (Doc2) (determined by SELEX; Figure 1D; Table S5). Closely related Tin and GATA motifs were also derived *de novo* from Tin and Pnr-bound sequences, respectively, by Junion et al. [26], whereas their Doc *de novo* motif deviates significantly from the SELEX-derived motifs for Doc (Figure 1D) and the related Tbx6 [37].

### Most tested Tin-bound regions act as enhancers in various mesodermal tissues

In a first step towards testing the observed Tin-bound regions functionally we ranked the binding peaks according to their size (as defined by the area underneath the contour), with the assumption that on average the regions bound more strongly (e.g., because of the presence of multiple binding sites, high affinity sites, or of binding sites for other Tin-binding factors that increase occupancy by Tin) would be more likely candidates for functional Tin targets [38]. As shown in the ranking lists for the 3–5.5 h and 5–8 h datasets of the top ∼450 peaks (Table S4A, S4B), the majority of known Tin downstream genes and proven Tin targets genes, defined previously via genetic and/or molecular data, were present among the genes associated with the top-ranking binding peaks. These included, in the “Early” (E) and “Late” (L) lists, respectively: *tup* (rank #E9, L34), *bap* (#E10, E198, L352), *mid* (#E19, L298, L350, L402, L411), H15 (#E22, L5), *mir-1* (#E43, E119, E155, L100), , *eya* (#E91, E128, L57, L165), *Doc* genes (#E105, E122, E282, L33), *Poxm* (#E138, L276), *zfh1* (#E141, L173, L227, L348), *mef2* (#E170, E181, L341), *jeb* (#E177), *odd* (#E81, L181; #E254, L164), *Six4* (#E255), *pnr* (#E261, L162), *slou* (#E273, E425, L140), *lbe* (#E308, L170), *Him* (#E318, L47), *Tl* (#E359), *disco* (#E420, L10, L217, L286), *btn* (#E455), *apt* (#L133, L336), *svp* (#L216, L330), *htl* (#L383), *lbl* (#L443), and *tin* (#L424). Apart from these, the top-ranking genes included many that are expressed in mesodermal tissues with patterns that overlap spatially and temporally with Tin (Table S4A, S4B).

For functional tests of enhancer activity *in vivo* we selected 51 binding regions (Table S4A, S4B). The following criteria were employed for the selection: 1) Higher priority was given to regions ranking higher on the size-ranked lists. 2) Lower priority was given to fragments that did not include at least one high-scoring Tin binding motif (Materials and Methods, Table S5). 3) Excluded were fragments associated with genes with published expression patterns that do not include mesodermal tissues. Thus, potential Tin targets in the Tin^+^ ectodermal foregut primordia were also omitted. The initial fragments had an average length of 1.4 kb and the endpoints were chosen such that the fragments were centered around the peak maxima and included any Tin motifs underneath the peaks.

**Figure 2:**
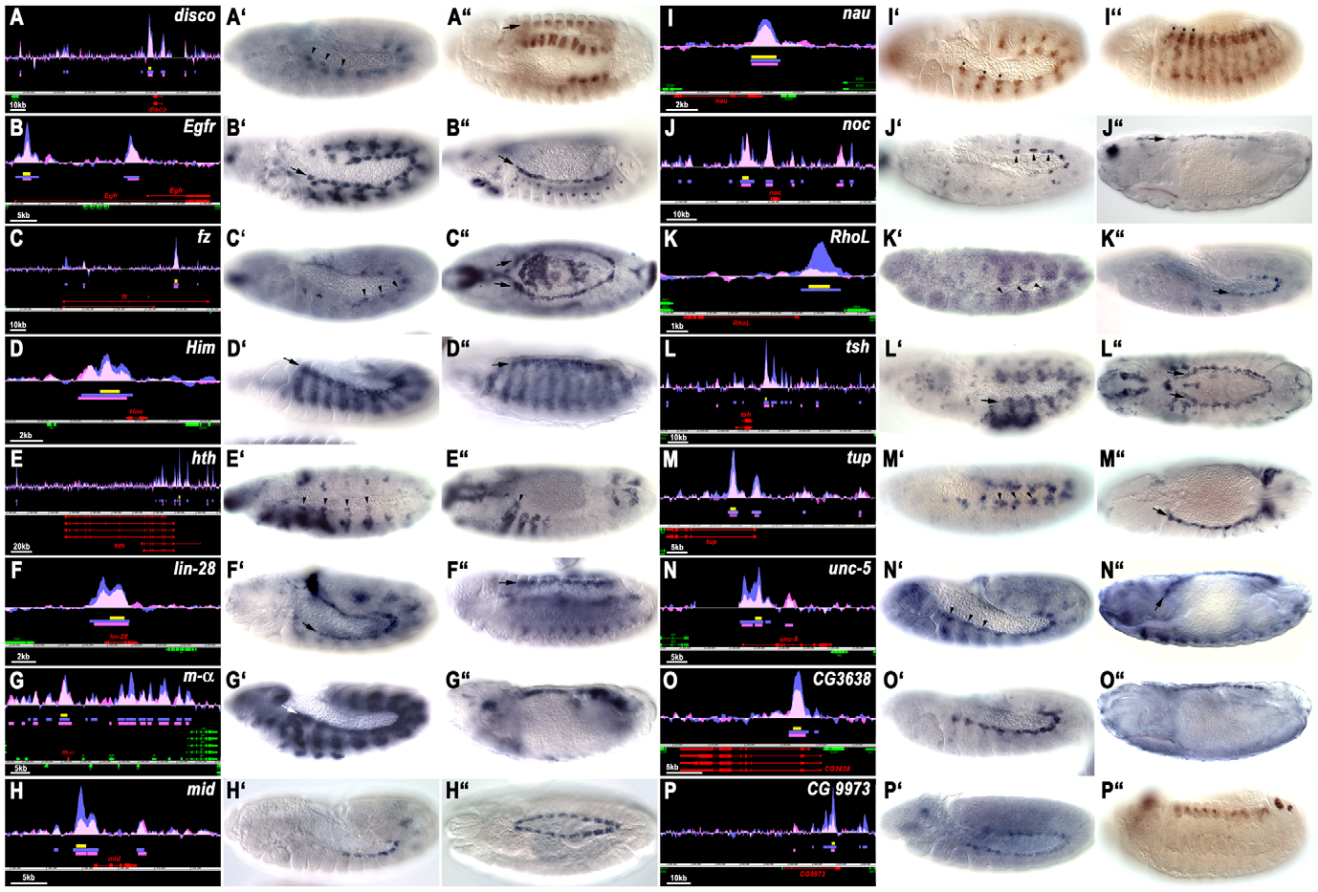
Tin binding and reporter activity of enhancers active in dorsal mesodermal cells, cardiac progenitors and heart progenitors. (A–P) show the Tin binding peaks (blue: 3–5.5 hrs: pink: 5–8 hrs AEL), the location of the tested enhancers (yellow bars) and the gene models (red: genes closest to tested fragment). Y axes are at identical scales whereas x axis scales are variable (see scale bars). A′ to P′ show enhancer activities at earliest stage of appearance (arrow or arrow heads: dorsal/cardiac mesoderm) and A″ to P″ at latest stage of detection in cardiac tissues (arrows or arrow heads). Shown are *GFP* or *lacZ in situ* hybridizations except in A″, I′, I″ and P″, which show anti-GFP antibody stainings (in A″, I″ and P″ perduring GFP from earlier expression). Genes are ordered alphabetically, with CGs last. (A′) Stage 12. Expression of *discoL10-GFP* in heart progenitors, segmental subsets of visceral muscle precursors and (A″), stage 14, perdurance of GFP in developing visceral muscles and heart. (B′, B″) Expression of *EgfrE1-lacZ* in cardioblast progenitors and subsets of somatic mesodermal cells (stage 12), and in cardioblasts (stage 14). (C′, C″) Expression of *fzL4-GFP* in cardioblast progenitors (stage 12) and in cardioblasts and amnioserosa (stage 14). (D′, D″) Expression of *HimL47-GFP* in cardiogenic mesoderm and somatic mesodermal cells (stage 12), and in cardioblasts and developing somatic muscles (stage 14). (E′, E″) Expression of *hthE54-GFP* in heart progenitors of Md, T1–T3 segments (arrow heads) and in somatic mesoderm (with A–P graded sizes of clusters) (stage 12) and in anterior developing heart cells (arrow head) and somatic muscles (stage 14). (F′, F″) Expression of *lin-28L64-GFP* in cardioblasts (stage 12, stage14). (G′, G″) Expression of *maL9-GFP* in cardiac and somatic mesoderm (stage 12) and in heart precursors (stage 14). (H′, H″) Expression of *midE19-GFP* in Tin^+^ cardioblasts (stage 12, stage 15). (I′, I″) Expression (I′, stage 12) and perdurance (I″ stage 14) of *nauL35*-GFP largely in dorsal somatic mesoderm (asterisks). (J′, J″) Expression of *nocL7-GFP* in pericardial cell progenitors (stage 12) and pericardial cells (stage 16). (K′, K″) Expression of *RhoLE102-GFP* in segmented dorsal mesoderm (stage 11) and cardioblast progenitors (stage 12). (L′, L″) Expression of *tshL8-lacZ* in cardiogenic mesoderm and thoracic ectoderm (stage 11) and in Tin^+^ pericardial cells (stage 15). (M′, M″) Expression of *tupE9-GFP* in cardiogenic mesoderm (stage 11) and cardioblasts (stage 14). (N′, N″) Expression of *unc-5L25-GFP* in cardioblast and pericardial cell progenitors (stage 12) and in cardioblasts and Tin^+^ pericardial cell (stage 16). (O′, O″) Expression of *CG3638L6-GFP* in cardioblast progenitors (stage 12) and cardioblasts (stage 15). (P′, P″) Expression of *CG9973E15-GFP* in cardiac mesoderm (stage 11) and perdurance of GFP in developing heart (stage 14).

39 of the 51 fragments tested (76%) were active in mesodermal domains overlapping with Tin domains. Among these, 16 (41%) were active in the cardiogenic mesoderm and/or in the developing heart, which were of particular interest to our study, and one (associated with *nau*) was active in the early dorsal somatic mesoderm (Figure 2; see Figure S2 for zfh1E141, which exhibits very weak activity in the dorsal vessel after stage 14; Tables S3A, S3B and S4). Several of these putative cardiac target enhancers of Tin were active additionally in cells of the somatic or visceral mesoderm (Figure 2; Tables S3A, S3B and S4). The onset of reporter expression within dorsal mesodermal and cardiogenic regions with these fragments included the time windows assayed in the ChIP experiments and the corresponding enhancers showed peaks in both the 3–5.5 h and the 5–8 h windows. Notably, several enhancers only began showing cardiac activity during the 5–8 h window, even though Tin binding was already detected during the 3–5.5 h window (Figure 2; lin-28L64, midE19, unc-5L25, CG3638L6) (note that the enhancers are named after the presumed associated gene and their highest rank in the Early and Late lists, respectively; Table S4). Conversely, other enhancers were still occupied by Tin in the 5–8 h window, but their activity in the dorsal mesoderm and developing heart had ceased during this period (Figure 2, discoL10, nauL35, CG9973E15, and data not shown). However, most of the cardiac enhancers remained active in developing cardioblasts and/or pericardial cells until stages 14–16 (Figure 2, EgfrE1, fzL4, HimL47, hthE54, lin-28L64, mαL9, midE19, nocL7, tshL8, tupE9, unc-5L25).

14 of the 39 positive fragments (36%) were active in the somatic mesoderm but not in the cardiogenic mesoderm (Figure S2). Half of these showed activity in the whole somatic mesoderm. Among these were fragments associated with *zfh1* (with additional weak expression in cardiac mesoderm), *CG5522*, *CG32792* and, surprisingly, *H15* and *hh*, two genes that are not known to be expressed in the somatic mesoderm. The other half showed activity in more restricted patterns in the somatic mesoderm, such as in large lateral cell clusters (lbeL170), segmental stripes (slpE35), and stripes restricted to ventral areas (PoxmE138, Six4E255).

6 of the positive fragments (15%) tested positive in the primordia of the trunk visceral mesoderm and one (oddE81) in fat body primordia (Figure S3). These included fragments near the genes *Alk*, *Fas3*, and *H2.0*, which are known to be expressed in the trunk visceral mesoderm. The enhancers of both *Alk* and *Fas3* start being active in the entire segmental visceral mesoderm primordia during the 3–5.5 h window (Figure S3A, S3B), whereas those of *Gukh*, *Nrt* and *H2.0* become active only during the 5–8 h window with their activity being restricted to the ventral rows of circular gut muscle founder cells (Figure S3C–S3E; wgnE1197-LacZ appears in portions of visceral mesoderm only at stage 14, Figure S3F). The remaining two positive fragments showed activity in the Dorsal Median (DM) cells, which form in the mesoderm along the ventral midline in each segment and later are attached dorsally to the CNS (Figure S4). The DM cells are known to depend on *tin* function, which must be needed during early stages when Tin expression is still present in ventral areas of the mesoderm (i.e., during our 3–5.5 h test window) [39]. In contrast to the above enhancer activities that overlap with Tin expression domains in the mesoderm, 12 additional enhancer fragments were active in a variety of non-mesodermal tissues and cell types that are mostly ectodermally-derived (Figure S5). A comparison with the genomic ChIP data published by Junion et al. [26] subsequent to our *in vivo* analysis shows that 50 of our 51 enhancer regions tested *in vivo* and all ten positive control enhancers used are also positive for Tin occupancy in their screens (the exception being the low ranking wgnE1197), and are often positive for various combinations of Doc, Pnr, pMad, and dTCF (Table S5). Perhaps unexpectedly, the 61 mesodermal and non-mesodermal enhancers can not be differentiated via their occupancy patterns by these factors, e.g., within each of the mesodermal (any tissue), cardiac, and non-mesodermal subset ∼1/3 of the enhancers are occupied by Tin+Doc+Pnr, ∼1/3 by Tin+Doc, and ∼1/3 by Tin only (Table S5). In addition, when we overlap our list of 61 enhancers with the ChIP data and predicted clusters of Junion et al., we find neither their individual ChIP signals nor any of their predicted ChIP cluster types to be significantly associated with cardiac enhancers (Fisher's exact test, P-value>0.10; a subtle association could have been missed because of the size of the dataset).

To get an overview of the involvement of *tin* in generating the observed patterns of mesodermal enhancer activity we tested a selection of the reporters from each class in *tin* mutant backgrounds. As shown in Figure S6A, S6A′, S6C, S6C′, S6H, S6H′, the enhancer activities of AlkE301, Fas3L254, and NrtL30 in the visceral mesoderm are severely reduced or absent in *tin* mutant embryos. Likewise, the activity of the *btn* enhancer in DM cells (Figure S6B, S6B′) and of the enhancers of *hth* (Figure S6D, S6D′, arrows) and *noc* (Figure S6G, S6G′) in cardiogenic regions is strictly *tin*-dependent. In contrast, the results for somatic mesodermal enhancers were more varied. Whereas the dorsal somatic mesoderm activities of the *nau* and *noc* enhancers were strictly dependent on *tin* (Figure S6F, S6F′, S6G, S6G′), the ventral somatic mesoderm activity of the *Six4* enhancer was reduced only slightly in *tin* mutants (Figure S6I, S6I′) and the somatic mesoderm activity of the *hth* and *zfh1* enhancers was completely *tin*-independent (Figure S6D, S6D′, S6J, S6J′). In summary, the selected fragments showing *in vivo* Tin binding were highly enriched for sequences containing enhancer activities in tissues with known genetic requirements for *tin*. Particularly the activities of the heart and visceral mesoderm enhancers appear to depend on *tin*, whereas the *tin*-dependency of somatic mesoderm enhancers seems less predictable.

### All tested enhancers active in early cardiogenic regions and in the mature dorsal vessel are *tin* dependent

**Figure 3:**
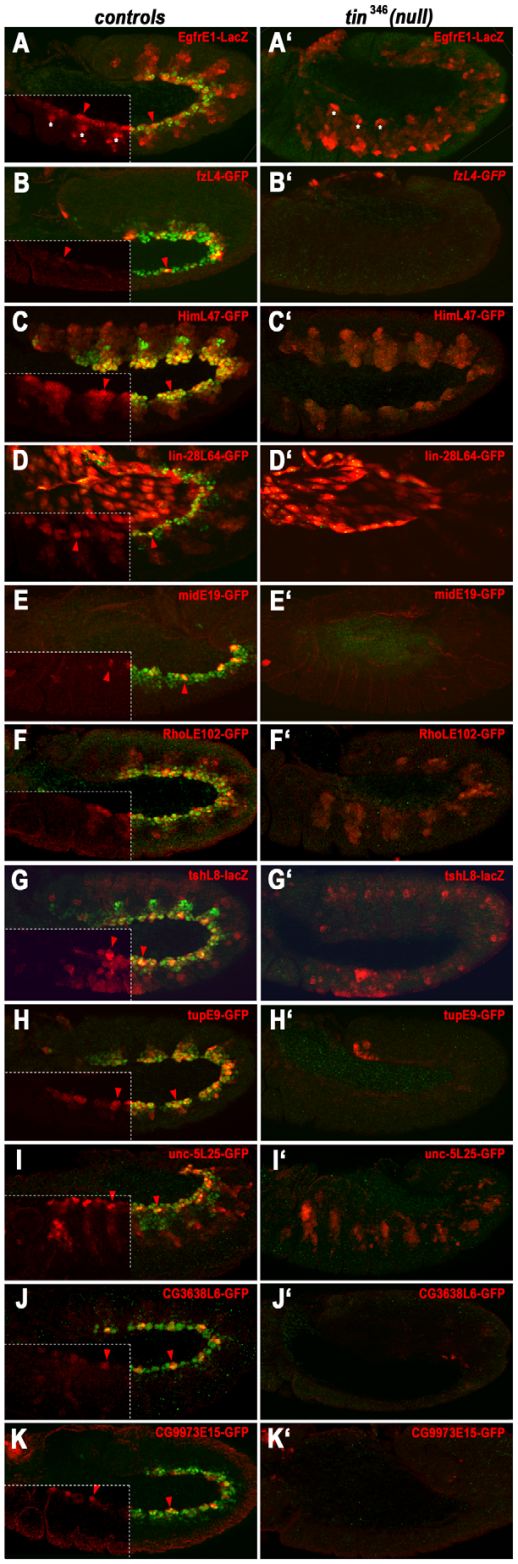
Dependency of early cardiac enhancer activities on *tin*. Shown are stage 11–12 embryos stained for enhancer activities (anti-βGal or anti-GFP) and Tin (green). (A–K) Enhancer activities in wild type backgrounds (left corner quadrants: anti-Tin omitted for better visualization of reporter patterns; arrow heads: early cardiac expression). (A′–K′) Enhancer activities in homozygous *tin* ^346^ mutant backgrounds. (A, A′) *EgfrE1*-LacZ expression in cardiac mesoderm but not in somatic mesoderm (asterisks) requires *tin*. (B, B′) *fzL4-*GFP expression in cardioblast progenitors requires *tin*. (C, C′) High-level *HimL47*-GFP expression in cardiogenic mesoderm requires *tin* but somatic mesodermal expression does not. (D, D′) *lin-28L64*-GFP expression in cardiac mesoderm requires *tin*. Amnioserosa expression is unaffected in *tin* mutants. (E, E′) *midE19*-GFP expression in cardioblast progenitors requires *tin*. (F, F′) *RhoLE102*-GFP expression in cardiogenic mesoderm, but not in somatic mesoderm, requires *tin*. (G, G′) *tshL8*-LacZ expression in cardiogenic mesoderm, but not in somatic mesoderm, requires *tin*. (H, H′) *tupE9*-GFP expression in cardiogenic mesoderm requires *tin*. (I, I′) *unc-5L25*-GFP expression in cardiac mesoderm but not in somatic mesoderm requires *tin*. (J, J′) *CG3638L6*-GFP expression in cardioblast progenitors requires *tin*. (K, K′) *CG9973E15*-GFP expression in cardioblast progenitors requires *tin*.

For the remainder of the study we concentrated on the characterization of the identified enhancers that were active in the cardiogenic mesoderm and developing heart. We verified that all of the enhancers with activities assigned to the cardiogenic mesoderm and heart progenitors during the developmental windows assayed by ChIP were indeed active in Tin-positive cells (Figure 3A–3J). The exact patterns varied, with some enhancers being active in all cells of the segmental cardiogenic anlagen (e.g., HimL47, tupE9, Figure 3C, 3H) and others becoming active shortly afterwards in newly-specified, segmentally arranged heart progenitors (e.g., fzL4, midE19, tshL8, unc-5L25, CG3638L6; Figure 3A, 3E, 3G, 3I, 3J) or throughout the cardiogenic mesoderm (EgfrE1, lin-28L64, Figure 3A, 3D). When assayed in *tin* null mutant backgrounds, the activities of all these enhancers in cardiogenic regions and heart progenitors were completely lost (Figure 3A′–3J′). By contrast, if enhancers were also active in other mesodermal areas, such as the somatic mesoderm (EgfrE1, HimL47, RhoLE102, unc-5L25; Figure 3A′, 3C′, 3F′, 3I′) or in amnioserosa and ectodermal tissues (lin-28L64, tshL8; Figure 3D′, 3G′), these non-cardiac activities did not require *tin*.

**Figure 4:**
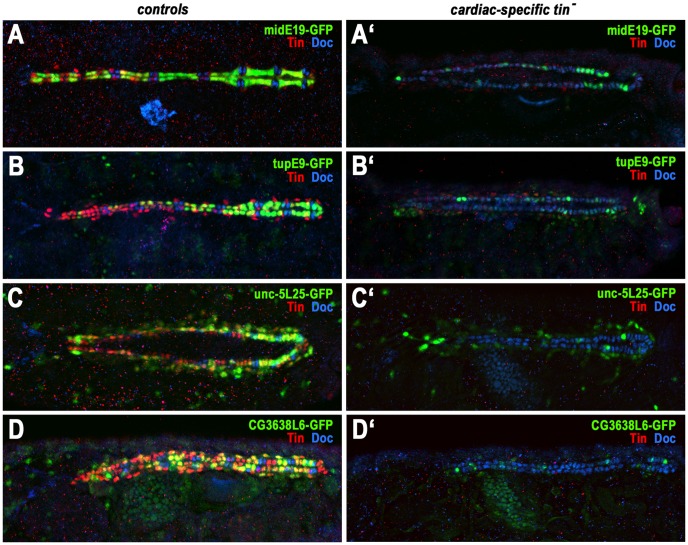
Dependency of late cardiac enhancer activities within the dorsal vessel on *tin*. Shown are reporter activities (anti-GFP, green), Tin^+^ cardioblasts and pericardial cells (anti-Tin, red) and Doc^+^ cardioblasts (anti-Doc, blue) in stage 15–16 control embryos (A–D) and in embryos specifically lacking Tin activity in cardiac cells (*tinABD*, *tin^346^*; A′–D′). (A) *midE19*-GFP is expressed specifically in the Tin^+^ cardioblasts. (A′) Absence of cardiac Tin expression causes a severe reduction of *midE19-*GFP activity. (B) *tupE9*-GFP is highly expressed in Tin^+^ cardioblasts (graded posteriorly-to-anteriorly) and, at much lower levels perduring from stage 12 expression, is present in Doc^+^ cardioblasts, pericardial cells, and dorsal somatic muscles. (B′) Upon loss of cardiac Tin expression almost all cardioblasts contain only low levels of perduring GFP. (C) *unc-5L25*-GFP expression in pericardial cells and (largely posteriorly) in cardioblasts. (C′) Absence of cardiac Tin expression causes near loss of cardioblast *unc-5L25*-GFP expression and a reduction of expression in pericardial cells. (D) *CG3638L6*-GFP is expressed in Tin^+^ cardioblasts (with variable intensities) and in Tin^+^ pericardial cells. (D′) Absence of cardiac Tin expression causes nearly complete loss of *CG3638L6*-GFP expression.

Whereas the activities of some of the assayed cardiac enhancers were transient and ceased by germ band retraction, the activities of several others persisted in the dorsal vessel until late stages of embryogenesis. The midE19 and tupE9 enhancers were active only in cardioblasts (especially in Tin^+^ cardioblasts) and the unc-5L25 and CG3638L6 enhancers in both cardioblasts and pericardial cells (Figure 4A–4D). To test whether these enhancer activities within cells of the dorsal vessel are also *tin* dependent we used a “conditional” *tin* mutant background, in which Tin is still expressed during early mesoderm patterning and heart progenitor specification but not in cardioblasts and pericardial cells after they are formed [9]. As shown in Figure 4A′–4D′, the activities of all four of these enhancers in cardioblasts (marked by Doc) and pericardial cells are nearly absent if Tin is not present in these cells. In summary, we found that all identified enhancers that drive expression in the cardiogenic mesoderm, heart progenitors, and cells of the dorsal vessel are strongly dependent on *tin*. Hence, these enhancers were strong candidates for direct functional targets of *tin*.

### Differential *in vivo* requirements for the binding motifs of the cardiogenic factors Tin, Pnr, and Doc

Next, we tested the *in vivo* relevance of the Tin binding sites in selected cardiac enhancers under investigation. Because the binding motif for the cardiogenic GATA factor Pnr had been prevalent among the genome-wide Tin-bound regions (Figure 1), and because the T-box factors Doc1-3 are known to have key functions during early cardiogenesis and cardioblast diversification as well [8], we also included the binding motifs of Pnr and Doc in this analysis. We note that the Doc binding motifs are closely related to the binding motifs for the Tbx20 cardiac T-box factors Midline (Mid) and H15 (Figure 1D). Thus, from stage 12 when these Tbx20 factors are expressed in cardioblast progenitors, the T-box binding motifs may be occupied either by Doc or Tbx20 factors.

**Figure 5:**
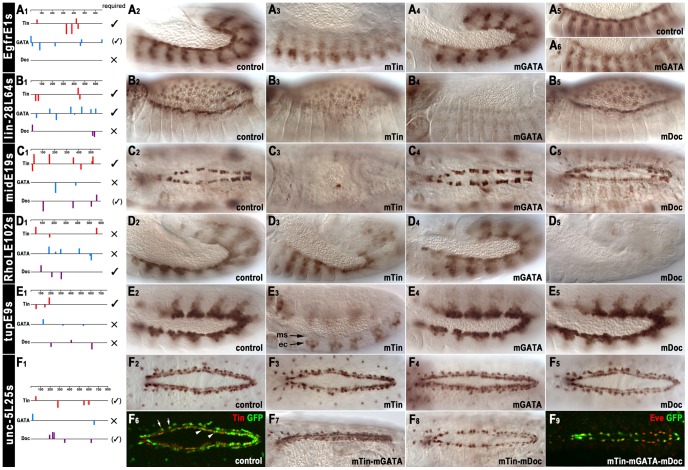
*In vivo* assays of the functions of predicted binding sites of Tin, Doc, and Pnr in identified cardiac enhancers. (A1–F1) Positions, orientations, and scores of predicted binding sites within cardiac enhancers (identical Y-scales). *In vivo* results for functionality of binding sites are summarized to the right (large ✓: essential; small (✓): required for full activity or redundantly with motifs for other factors; × not required). (A2, A3) *EgfrE1s*-GFP activity in cardiac mesoderm (A2, stage 12) is lost upon mutation of Tin binding motifs shown in (A1). (A4–A6) Mutation of GATA motifs shown in (A1) leads to a mild reduction of *EgfrE1s*-GFP activity at stage 12 (A4) and a more significant reduction at stage 14 (A6). (B2, B3) *lin-28L64s*-GFP activity in cardioblasts (B2, stage 14) is lost upon mutation of Tin binding motifs. (B4) Mutation of GATA motifs leads to loss of *lin-28L64s*-GFP activity in both cardioblasts and amnioserosa cells. (B5) Mutation of Doc binding motifs affects neither cardioblast nor amnioserosa activity of *lin-28L64s*-GFP. (C1, C2) *midE19s*-GFP activity in cardioblasts (C2, stage 15) is lost upon mutation of Tin binding motifs (C3). (C4) Mutation of GATA motifs does not affect *midE19s*-GFP activity. (C5) Mutation of Doc binding motifs reduces cardioblast *midE19s*-GFP activity. (D2–D4) Mutation of the binding motifs for Tin (D3) or Pnr (D4) does not affect *RhoLE102s*-GFP activity in the cardiogenic and dorsal somatic mesoderm. (D5) Mutation of Doc binding motifs causes loss of *RhoLE102s*-GFP activity in cardiogenic and dorsal somatic mesoderm. (E2, E3) Mutation of Tin binding motifs causes loss of *tupE9s*-GFP activity in dorsal and cardiogenic mesoderm (ms). Ectopic *tupE9s*-GFP occurs in dorsal ectoderm (ec). (E4, E5) Mutation of GATA motifs (E4) or Doc binding motifs (E5) does not affect *tupE9s*-GFP activity. (F2–F5) Mutation of either the Tin binding motifs (F3), the GATA motifs (F4) or the Doc binding motifs (F5) does not affect *unc-5L25s*-GFP activity in cardioblasts and pericardial cells at stage 15. Three sequences, CCAAGGG, TCAATTG, TCGAGTG, poorly matching the Tin binding motif (Figure 1, Table S5), are still present but it is unknown whether they can bind Tin. (F6) Staining of stage 15 *unc-5L25s*-GFP control embryo for GFP and Tin identifies GFP-positive cells as cardioblasts (arrow heads) and Tin^+^ pericardial cells (arrows). (F7) Simultaneous mutation of Tin binding motifs and GATA motifs does not affect cardiac *unc-5L25s*-GFP activity. (F8) Simultaneous mutation of Tin and Doc binding motifs severely reduces *unc-5L25s*-GFP activity in cardioblasts but not in pericardial cells. (F9) Simultaneous mutation of binding motifs for Tin, Pnr, and Doc abrogates *unc-5L25s*-GFP activity (anti-GFP, green) in cardioblasts but not in pericardial cells (anti-Eve, red).

Figure 5A1–5F1 shows the distribution of the Tin, Pnr, and Doc binding motifs within the cardiac enhancers from *Egfr*, *lin-28*, *mid*, *RhoL*, *tup*, and *unc-5*. The Tin matrix from proven target genes (Figure 1B), the SELEX-based matrix from the Pnr-related vertebrate cardiogenic factor GATA-6 with GATA core sequence [40], and our SELEX-based Doc2 motif (Figure 1D) were used to locate and score binding motifs. Subsequent to our functional analysis, Junion et al. [26] found regions overlapping with our enhancers from *Egfr*, *lin-28*, *RhoL*, and *unc-5* to be occupied by all three cardiogenic factors, whereas the *mid* and *tup* enhancers appeared to be solely occupied by Tin (Table S5). For all six chosen enhancers, shortened versions (∼600–800 bp) were used for functional analysis of binding motifs, named EgfrE1s, lin-28L64s, midE19s, RhoLE102s, tupE9s and unc-5L25s (Tables S4, S5). All enhancers examined included binding motifs for all three cardiogenic factors except for the EgfrE1s enhancer, which lacked Doc motifs. The numbers and spatial arrangements of these motifs within the different enhancers were quite variable (Figure 5A1–5F1).

In the EgfrE1s enhancer, the Tin motifs were essential for enhancer activity in the cardiogenic mesoderm, but not in the somatic mesoderm (Figure 5A3, cf. Figure 5A2). Mutation of the GATA (Pnr) motifs caused a weakening of the cardiac versus somatic mesodermal enhancer activity at early stage 12 (Figure 5A4), which became even more pronounced during stage 13 (Figure 5A6, cf. Figure 5A5).

In the lin-28L64s enhancer, the Tin motifs were essential for cardioblast activity, and the GATA sites were essential for both cardioblast and amnioserosa activity (Figure 5B3, B4, cf. Figure 5B2; note that Pnr expression includes both the cardiogenic mesoderm and the amnioserosa, as well as the dorsal ectoderm). By contrast, the Doc motifs were not essential for lin-28L64s enhancer activity (Figure 5B5).

In the midE19s enhancer, the Tin motifs were essential for its activity in cardioblasts (Figure 5C3, cf. Figure 5C2, see also [19]). Mutation of the GATA motifs did not have any effects, but mutation of the Doc motifs led to a strong reduction of enhancer activity in the Tin^+^ cardioblasts (Figure 5C5). It is conceivable that this effect is caused not only by the failure to bind Doc during heart progenitor specification but also by the inability to bind Mid, which may prevent maintenance of activity by autoregulation (in combination with Tin) in developing cardioblasts. A second effect of the Doc-site mutations was a moderate upregulation in the Tin^−^/Doc^+^ subset of cardioblasts, in which this particular enhancer is normally not active although *mid* is expressed there. Thus, the combined effects of the Doc site mutations lead to weak uniform enhancer activity in all cardioblasts. These observations point to an unexpected complexity of *mid* regulation through both positive and negative effects of cardiogenic regulators and to the existence of yet unidentified regulatory elements for expression in the Tin^−^/Doc^+^ cardioblasts.

The RhoLE102s enhancer was active in the cardiogenic mesoderm even after mutation of any decent Tin motifs, which was unexpected (Figure 5D3, cf. Figure 5D2). Although one low-scoring sequence motif, CCAAGGG is still present in the mutated version, this particular sequence deviates significantly from the motifs shown in Figure 1A and is not known to bind Tin *in vitro*. Mutations of the GATA motifs also did not have any effects (Figure 5D4). However, in the absence of the Doc motifs the RhoLE102s enhancer was inactive (Figure 5D5).

In the tupE9s enhancer, only the Tin motifs were required for its activity in the cardiogenic mesoderm, whereas the GATA and Doc motifs were not essential (Figure 5E3, cf. Figure 5E2, 5E4, 5E5). These results are compatible with the absence of *in vivo* binding by Pnr and Doc [26]. Upon mutation of the Tin binding sites, enhancer activity in the dorsal mesoderm was completely lost and instead, ectopic segmental activity in the dorsal ectoderm appeared. Of note, a similar switch in germ layer activities has also been observed upon mutation of the dorsal mesodermal enhancers of *tin* and *bap*. For these two examples, it was found that Tin acts synergistically with Smads bound to nearby sites during the activation of the enhancers in dorsal areas of the mesoderm. By contrast, in the dorsal ectoderm an unknown repressor with a recognition site that overlaps with the Tin motif was proposed to bind and prevent ectodermal enhancer activation by Dpp and activated Smads [7]. The *tup* enhancer appears to be a third example for this regulatory mechanism to achieve germ layer specificity of the Dpp response.

Surprisingly, for the unc-5L25s enhancer neither mutation of the Tin motifs nor of the GATA or Doc motifs had any measurable effect on its activity in cardioblasts and pericardial cells (Figure 5F3, 5F4, 5F5, cf. Figure 5F2). We can not exclude that Tin can still bind and provide full activity through three low-scoring candidate Tinman motifs that were not mutated (see Figure 5, legend). Alternatively, or in addition, there is the possibility of functional redundancy among these three factors and their binding sites within this enhancer, which we tested with derivatives containing combinatorial mutations in the different motifs. When the Tin and GATA motifs were mutated in combination, there was still no change in the cardiac activity of the enhancer (Figure 5F7). By contrast, simultaneous mutation of both the Tin and Doc motifs caused near absence of enhancer activity in cardioblasts, whereas its activity in pericardial cells was unaffected (Figure 5F8). Additional mutation of the GATA motifs had only minor additive effects on the reduction of enhancer activity, which showed only residual expression in Eve^+^ pericardial cells (Figure 5F9, cf. Figure 5F6, 5F8). Hence, we propose that in the case of the *unc-5* enhancer, binding of either Tin or Doc (and perhaps Mid) to the Tin or T-box motifs, respectively, is functionally relevant but either factor alone is largely sufficient for activating the enhancer.

### *De novo* motif discovery of a sequence motif enriched in cardiac enhancers

**Figure 6:**
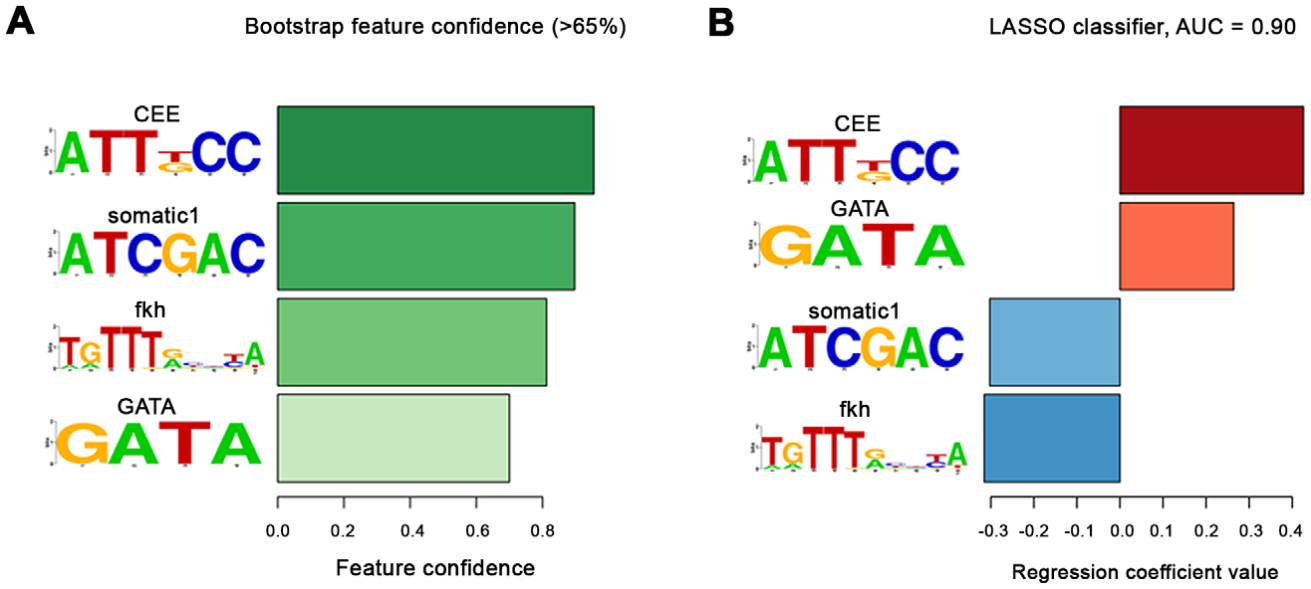
Cardiac enhancers classifier. (A) The motifs with bootstrap confidence over 65%. The confidence levels are shown as bars and are colour-coded with darker color for larger confidence. The motif with the greatest confidence was the *de novo* CEE motif followed by another *de novo* motif somatic1. The GATA motif and the fkh motif from JASPAR had smaller confidence. As expected, Tin motifs were not retrieved as all fragments had been selected based upon high Tin occupancy, which in most cases involves Tin motifs, whereas the classifier was trained to distinguish between the two groups of enhancers. (In addition, the exact borders of both the cardiac and non-cardiac enhancers had been adjusted for their inclusion of one or more Tin motifs, if present within the binding peak region). (B) The regression coefficients in the final classifier that contains only the 4 motifs with >65% confidence. During classifier training the cardiac enhancers were given a target value of 1 and the rest of enhancers −1. Enhancers are scored by multiplying the regression coefficients with the standardized motif hit density (standardized to zero mean and unit variance over the whole dataset) in the sequence. Thus, a positive regression coefficient indicates that the above-average motif presence predicts cardiac enhancers, and negative that it predicts non-cardiac enhancers. The motif with the largest positive regression coefficient is the CEE motif followed by GATA. GATA (Pnr) is a known co-factor of Tin, which we further verified in our mutation analysis, while CEE was the novel predicted motif (see main text). Presence of two motifs: somatic1 and fkh predicted the non-cardiac enhancers. Somatic1 is another *de novo* motif we discovered (see Materials and Methods) but which we did not test functionally, while fkh indicates that a protein from the forkhead family likely binds to those Tin-bound enhancers that are not active in the cardiac cells but in other parts of the mesoderm. Note that the AUC score shown is an overly optimistic estimate of real generalization error due to selection bias [58].

We used machine learning methods to test whether we can predict cardiac enhancers based on their motif content, as had previously been shown for human heart enhancers [74]. For this analysis, we added ten well-characterized Tin-dependent enhancers that are active in cardiogenic tissues, visceral mesoderm, or somatic mesodermal cells to the collection described herein, which yielded 24 cardiac enhancers and a total of 61 enhancers to be examined (Table S5). The enhancers were classified according to their tissue-specific activity patterns as denoted in the above table. First we undertook extensive *de novo* motif discovery using RSAT [32] on each experimentally verified enhancer class separately and between pairs of enhancer classes. This yielded 33 *de novo* motifs, which we put together with previously described motifs in *Drosophila* (see Materials and Methods). To prioritize motifs predictive in cardiac enhancers we constructed a LASSO classifier to predict the 24 cardiac enhancers from the set of 61 experimentally verified enhancers (Narlikar et al., 2010). Since our set of enhancers is small and we wanted to assign confidence values to predictive motifs, we used a bootstrap modification of LASSO called bolasso [41]. We found one *de novo* motif to be especially predictive of the cardiac class, ATT[TG]CC, which we termed “Cardiac Enhancer-Enriched (CEE) Motif”. This motif has a bootstrap confidence of 96% and its presence is the strongest predictor of cardiac enhancers followed by the GATA motif (Figure 6). In several sequences with cardiac activity, the CEE motif is particularly frequent, namely in midE19 (6.9 motif hits per kb), mef2 I-E (5.3 per kb), mef2 II-B[S] (4.3 per kb) [42], lin-28L64 (4.2 per kb), hthE54 (4.1 per kb), zfh1E141 (4.1 per kb) and sli (3.8 per kb). The enhancers with cardiac activity have an average density of 2.86 CEE motif hits per kb. In contrast, the experimentally verified enhancers with no cardiac activity have the average density of 1.52 per kb of sequence, which is almost identical to that in 2 kb *Drosophila* gene upstream regions (1.53 CEE motif hits per kb).

**Figure 7:**
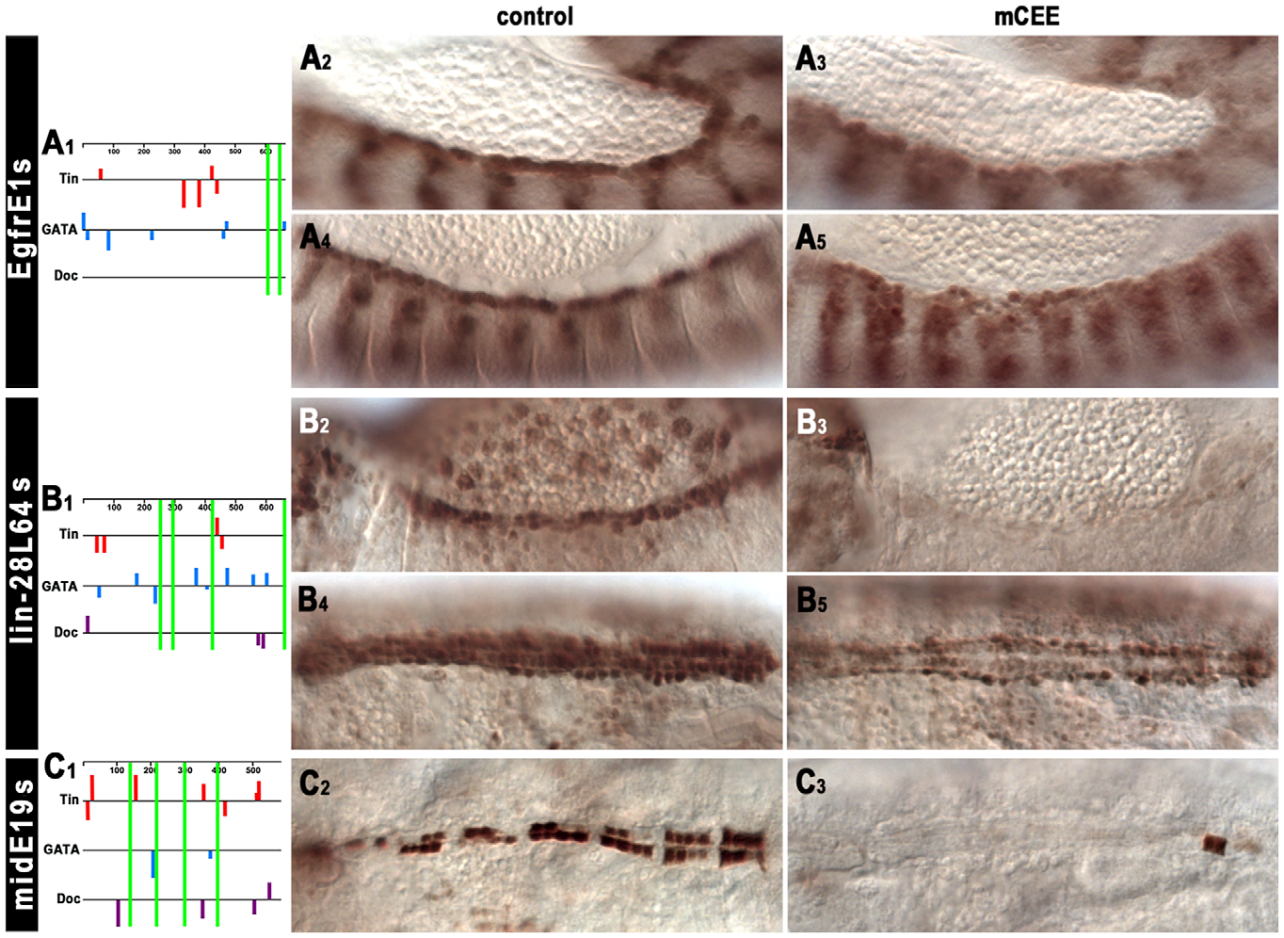
*In vivo* assays of the function of the CEE motifs in selected cardiac enhancers. (A1, B1, C1) Schematic drawings of the predicted Tin, Pnr, and Doc binding sites within cardiac enhancers as in Figure 5 and the positions of the CEE motifs relative to these (green bars). (A2–A5) Mutation of the CEE motifs causes a strong reduction of *EgfrE1s*-GFP activity in cardioblast progenitors at stage 12 (A2, A3) and in cardioblasts at stage 14 (A4, A5). (B2–B5) Mutation of the CEE motifs leads to the absence of *lin-28L64s*-GFP activity in heart precursors (and amnioserosa cells) at stage 14 (B2, B3) and a reduced activity in cardioblasts and pericardial cells at stage 16 (B4, B5). (C2, C3) Mutation of the CEE motifs causes almost complete loss of *midE19s*-GFP activity in Tin^+^ cardioblasts (stage 16).

Because of the relatively small size of the enhancer dataset we needed to use all of the data for *de novo* motif finding and model selection, which can lead to an over-estimation of the AUC. For this reason, it was important to test the predictions of the classifier (namely the CEE motif) for their *in vivo* relevance in transgenic animals carrying reporter constructs in which the CEE motifs were mutated. We included all enhancer fragments that were also tested for functionality of the Tin, Doc, and GATA motifs and in addition, the published cardiac *mef2* enhancers I-E and II-B[S]. As shown in Figure 7, in three of these fragments the CEE site mutations caused severe reductions of their cardiac enhancer activity. In EgfrE1s, enhancer activity in heart progenitors at stage 12 (Figure 7A3, cf. Figure 7A2) and particularly in cardioblasts following stage 13 (Figure 6A4, cf. Figure 6A3) was strongly reduced, whereas the activity in the somatic mesoderm was unaffected. In lin-28L64s, CEE site mutation caused loss of enhancer activity in cardiac cells in embryos until stage 14 (Figure 7B3, cf. Figure 7B2). Activity in the amnioserosa was also lost, whereas head expression was unaffected. In the maturing dorsal vessel, the CEE site-mutated lin-28L64s enhancer regained some activity, particularly in pericardial cells, but its activity in cardioblasts remained much lower than that of the unmutated control (Figure 7B5, cf. Figure 7B4). For the midE19s enhancer, CEE site mutations caused a complete loss of activity in the vast majority of Tin^+^ cardioblasts. In rare remaining positive cardioblasts, nearly full activity was seen (Figure 7C3, cf. Figure 7C2). These results were very similar to the results obtained with Tin site mutations for these three enhancers, and for EgfrE1s and lin-28L64s also with the GATA site mutations, even though the CEE motifs do not overlap with any of the Tin and GATA motifs (Figure 7A1, 7B1, 7C1). Altogether, we hypothesize that the CEE motifs bind a yet unknown co-factor of these cardiogenic factors. At least in the cases of EgfrE1s, lin-28L64s, and midE19s, the combination of this co-factor and one or several of the cardiogenic factors is needed for activating the respective enhancer in cardiac cells. Although the verification rate of three functional CEE motifs out of eight may seem modest, it is in the same range as found for the Doc motifs (2/5) and GATA motifs (2/6). It remains to be shown whether this factor is not functional in the other five enhancers tested, in which CEE site mutations did not cause any noticeable differences in cardiac activity. Alternatively, it is conceivable that in the negative cases additional, degenerate versions of this sequence motif were present and functional, or that there are additional binding sites for different co-factors that function redundantly with the putative ATT[TG]CC binding factor.

## Discussion

Although the developmental functions of *tin* during mesodermal tissue and particularly heart formation have been defined in quite some detail by genetic approaches, it is far from being clear how many target genes of *tin* are involved in executing these functions. Candidate approaches based on genetic observations have led to the identification of a relatively small number of essential Tin downstream targets, most of which correspond to members of the transcriptional network controlling the development of the heart, visceral muscles, and specific body wall muscles. The chromatin immunoprecipitation approach taken in our present study, as well as in studies performed in parallel by others [24], [25], provides a picture of the upper limit of potential Tin target genes that could be relevant biologically, although this number still depends on the specific cut-off used. With this latter approach, the challenge is to determine the particular fraction of genes associated with Tin binding *in vivo* that indeed require *tin* and are utilized for implementing the various *tin* dependent programs of mesodermal tissue development. In our present study we have begun to address this issue with a combination of genetic tests and enhancer dissections.

### Global aspects of Tin occupancy and the question of functionality of binding

A comparison between the Tin-bound regions from our study and that of Zinzen et al. [25], which were done using very similar developmental time windows, shows strong overlaps. In addition, almost all enhancers known to be targeted directly by Tin during the tested time windows and the large majority of known *tin* downstream genes were found to be associated with *in vivo* Tin binding. The overall similarity of binding data obtained in different labs corroborates that a majority of reported peaks reflects authentic *in vivo* occupancy although there can be differences in sensitivities. For example, the number of Tin-peaks in our early window was ∼2.5 times larger than that in the early time window from Zinzen et al. [25], which we partially attribute to our use of affinity-purified anti Tin.

The observed prevalence of the GO terms “mesoderm development” and “heart development” and the prevalence of genes associated with Tin occupancy with expression patterns in “trunk mesoderm primordium”, “visceral mesoderm primordium”, and “cardiac mesoderm primordium” (BDGP; [28]) underscores the notion that Tin is bound to a large number of enhancers that are active in tissues requiring *tin*. This was further confirmed by the activity of ∼3/4 of our reporter constructs in various mesodermal domains that overlap with the presence of Tin protein (although this number may be somewhat skewed because we purposely omitted Tin-occupied sequences near known non-mesodermal genes in our analysis). Comparable results were also reported by the Furlong group [24]–[26]. Although at first glance, this could be taken as an indication that Tin is a direct regulator of most of these tested enhancers and globally-bound elements, there is an increasing body of evidence suggesting that *in vivo* binding of most factors is very promiscuous. According to this view (e.g., Biggin (2011) [43]), the high concentrations of nuclear Tin could simply drive binding to sequences containing its cognate binding motifs, as long as they are accessible as would be the case in mesodermally-active enhancers. Indeed, several observations indicate that many Tin-bound sequences do not function as Tin-dependent enhancers. For example, genes expressed in ectodermal including neuronal tissues but not in the mesoderm were also very prevalent globally among the genes linked to Tin-occupancy, and clearly are not activated by Tin. A prominent example of a gene with ectodermally-restricted expression associated with Tin-binding is *vnd*, which is flanked by one of the most highly Tin-occupied sequences in the entire genome. Of note, it has been shown that in mesodermal cells this particular region features the presence of repressing chromatin marks (H3K27me3) and the absence of activating marks and of PolII, but contains H3K4me1 which frequently marks potential enhancers [44]. This situation contrasts with the one found for most enhancers active in the mesoderm, which tend to show low or absent H3K27me3 but high activating marks and PolII. One possible interpretation could be that at the *vnd* locus, Tin functions in maintaining the repressed state of a potential enhancer and as a consequence excludes *vnd* expression from the mesoderm in order to restrict it to the ventral ectoderm (perhaps in cooperation or temporal succession with the Snail repressor [45]). In certain contexts *tin* does have repressing functions, particularly during the regulation of *Doc* within the dorsal vessel, although the mode of action of Tin in this situation is not yet known [9], [46]. Likewise, Tin might repress other ectodermal enhancers in the mesoderm, but genetic evidence for such a role of *tin* is currently lacking. An alternative explanation for Tin binding to *vnd* sequences would be that it is opportunistic and does not have any functional consequences. As both Tin and Vnd belong to the NK homeodomain family and share closely related binding motifs, Tin may associate neutrally with NK homeodomain motifs in the mesoderm that are actually destined for binding of Vnd and *vnd* autoregulation in the ectoderm, if they are accessible despite inactivating chromatin marks [47], [48]. Analogous situations could exist at other ectodermal target genes of *vnd* and related NK homeobox genes (e.g., Scarecrow [49]). For a deeper understanding of the genome-wide roles of Tin-associated sequences it will be necessary to include ectodermally-expressed genes linked to Tin-binding in functional dissections to distinguish between neutral and potentially repressive binding.

A significant portion (>20%) of the Tin-bound regions overlap with so-called HOT regions, which are characterized by the simultaneous binding of >8 different transcription factors during pregastrulation stages [27]. It was shown that the vast majority of HOT regions have enhancer activity, although these do not necessarily reflect the patterns and stages predicted by the factors bound to them during blastoderm stages [50]. It is thought that binding of ubiquitous DNA-binding proteins, particularly the TAGteam factor Zelda and the GAGA factor (Trithorax-like), to their binding motifs within HOT regions establishes open chromatin and that mass action then attracts a variety of other factors, which in many cases bind neutrally [50]; reviewed in [51]. The observation that many HOT regions can later serve as Tin-bound mesodermal enhancers may suggest that these enhancers are “primed” by the binding of chromatin-opening factors to facilitate binding of mesodermal factors during post-gastrulation stages. Again, in some cases mesodermal factors including Tin may bind functionally whereas in other cases they may bind neutrally. In agreement with this proposal, ∼50% of the Tin-bound mesodermal enhancers from our study overlapped with HOT regions. Seven among these (tupE9, unc-5L25, malphaL9, CG3638L6, EgfrE1, eveE428, sliL427) showed cardiac expression patterns and the others were active in the somatic and/or visceral mesoderm. In every case among the above HOT regions with cardiac activities where tested, Tin binding was shown to be functional.

The sizable yield of cardiac enhancers with functionally important Tin binding sites is consistent with the known key role of *tin* during various stages of cardiac development. In addition, in light of global data from other factors and the distributions of their binding motifs [38], [50], our exclusion of regions with low levels of Tin binding and without well-matching Tin binding motifs likely selected against regions with promiscuous and neutral Tin binding. Nevertheless, we did encounter several examples of Tin-bound mesodermal enhancers that were still active in *tin* mutant backgrounds, particularly among those active in the somatic mesoderm. In some cases shown herein, as well as in the case of the Fas3L254 enhancer (H.J. and M.F., unpublished), mutations of the Tin binding motifs did not affect enhancer activity in the somatic, cardiac, or visceral mesoderm, respectively. We infer that also at mesodermal enhancers, transcription factors such as Tin can bind neutrally, for example if a fortuitous binding motif is made accessible by other factors, including “chromatin-opening” factors or specific histone marks. In other instances, they may be attracted via mass action solely involving protein-protein interactions, without significantly contributing to the enhancer activity. Unfortunately, laborious tests are necessary in each case to firmly distinguish between different scenarios such as, 1) binding that is biologically essential; 2) binding that makes subtle contributions to the enhancer activity, which may either be biologically irrelevant or perhaps affect the robustness of the developmental process; 3) binding that is functionally redundant with that of other factor(s); 4) promiscuous binding with neutral effects due to the absence of necessary co-factors; 5) binding that is functionally neutralized by a co-bound repressor, as has been shown in the case of some visceral mesoderm enhancers that bind both Tin and the repressor Sloppy paired (Slp) [26]. More generally, it is becoming apparent that, in the absence of functional *in vivo* tests, global binding data and computational enhancer predictions need to be interpreted with great caution (as for example discussed in [43], [52], [53]). Thus, we propose to reserve the terms “target” and “CRM” for the sequences that have been verified as such *in vivo*.

In our present study we have performed such tests with a select number of Tin-bound enhancers active in the cardiogenic mesoderm and heart and also included the binding motifs of two other major cardiogenic factors, Pnr and Doc. In five of the six tested enhancers we could demonstrate that Tin binding is functional. This result suggests that, at least among enhancers with cardiac activity, high Tin occupancy, and well-matching Tin binding motifs, there is a high probability that Tin binding is functional rather than being promiscuous.

### The role of combinatorial occupancy of cardiac enhancers by cardiogenic factors

Each of the six dissected enhancers showed distinct properties with regard to its dependency on the binding sites for the different cardiogenic factors and featured very diverse spatial arrangements of these sites. Thus, EgfrE1s showed complete dependency on the Tin sites, but depended on Pnr sites only from stage 13 onwards and lacked any Doc sites. lin-28L64s required both Tin and Pnr sites independently, but not the Doc sites. midE19s required both the Tin and the Doc sites, but not the Pnr sites. RhoLE102s required the Doc sites but, surprisingly, not the Tin sites and also not the Pnr sites. tupE9s required only the Tin sites but not the Pnr and Doc sites. Finally, in unc-5L25s none of the sites of the three cardiogenic factors were required individually, but in cardioblasts the presence of either Tin or Doc sites (and, to a lesser degree, Pnr sites) was essential, which suggests full or partial functional redundancy between these factors when bound to this enhancer. In pericardial cells, enhancer activity was retained in the total absence of binding sites for all three factors, as was the case in cardiac-specific *tin* mutants. It is likely that in the wild type, early *tin* activates a different set of transcription factors in these pericardial cells, which in turn activate the *unc-5* enhancer. Altogether, our findings illustrate the diverse functional architecture of each of these cardiac enhancers. At least in part, this reflects their diverse temporal, spatial, and cell type-specific activity patterns. Clearly, a lot more effort will be required before we can fully explain the differential requirement for individual factors and correlate it with the specific activity patterns of the respective enhancers.

In their recent study, Junion et al. [26] assayed global *in vivo* binding of Pnr and Doc, as well as the Dpp and Wg signaling effectors Mad and TCF, and compared their occupancies with that of Tin. They showed that a significant fraction (∼20–40%, depending on the cut-off) of the Tin-bound regions were co-occupied by all four of the other factors, which reinforces the notion that factors are often co-recruited to active enhancers in the tissues in which they are jointly expressed [43]. An analogous situation was found for the global occupancies of Nkx2-5, GATA4, and Tbx5 in a mouse cardiomyocyte cell line and of Nkx2-5, GATA, and Tbx3 in adult mouse heart, albeit with a lower degree of overlap as compared to *Drosophila* [54], [55]. The large majority of the tested *Drosophila* enhancers of this type were indeed active in the dorsal and cardiogenic mesoderm, in which these factors overlap [26]. While these authors did not examine the functionality of individual binding motifs in these enhancers, our present study suggests that for most enhancers the binding of only one or two cardiogenic factors among the three (Tin, Pnr, Doc) is essential and sufficient. Regardless of whether binding is functional or not, the tendency of co-recruitment of factors to active enhancers and the knowledge of their domains of spatial overlap facilitates machine learning approaches to predict spatial patterns of enhancer activities [25].

Largely based on their analysis of the global frequencies of the motifs for Tin, Pnr and Doc within the regions binding all five tested factors versus in those binding only Tin plus one other factor, Junion et al. [26] proposed that Pnr and Doc are recruited preferentially to these enhancers to promote the subsequent recruitment of the other factors including Tin. Although we agree that examples for such an activity of Pnr and Doc might exist, the global data may not be sufficiently reliable to deduct such a general rule. Moreover, the particular enhancer used to support this model appears to be active exclusively in ectodermal stripes and not in the mesoderm (which is consistent with the RNA expression pattern reported for the associated gene, *CG14888*; BDGP; [28]). Instead, the initial pan-mesodermal expression of Tin, which occurs prior to the dorsal mesodermal expression of Pnr and Doc and is needed upstream of *pnr*, may argue for the possibility that it is more commonly Tin rather than Pnr and Doc that functions like a pioneer to promote the recruitment of subsequent cardiogenic factors. Tin may be bound to co-repressors prior to the recruitment of subsequent activators, which could sharpen the on/off state of enhancers [46], [56]. In the future, these models can be tested via assaying the *in vivo* occupancies of cardiogenic factors on binding site-mutated enhancers or, globally, the occupancies of embryonic enhancers in purified mesodermal cells from *tin*, *pnr*, or *Doc* mutant embryos.

### The role of the newly discovered CEE motif in cardiac enhancers

The CEE motif discovered by our machine learning approach using 24 enhancers with cardiac activities vs. 37 non-cardiac enhancers was the motif most highly predictive for the cardiac set of enhancers. The validity of the approach was supported by the fact that we also found the GATA motif as a classifier for cardiac enhancers whereas the forkhead domain binding motif was found as a classifier for somatic mesodermal enhancers. The latter was also reported in a recent study using a related approach [57]. It should be noted that the *de novo* CEE motif is both constructed on and used for the classification of the same dataset. This can lead to selection bias that can produce both a high false positive rate in discovering significant motifs and an overly optimistic estimate of the classifier performance [58]. Therefore, experimental validation of *de novo* motifs prioritized by the machine learning approach is required to verify their biological function.

In light of these caveats, our findings that three out of eight tested enhancers require the CEE motifs for normal cardiac activity do support the conclusion that these motifs correspond to binding sites of a crucial factor in cardioblasts and their progenitors. This putative factor likely cooperates with the other cardiogenic factors that are active at these enhancers. We can not exclude that this factor is also active when bound to the other cardiac enhancers that did not depend on its binding sites, where it may be functionally redundant. Based upon the sequence of the CEE motif it is difficult to predict the identity of the corresponding binding factor with confidence. However, we note that the CEE motif bears a striking similarity with the binding motifs of several vertebrate Ets domain factors (e.g., FEV, SPI1; [59]). In *Drosophila*, the Ets domain factor Pointed (Pnt) has been identified as an important regulator of heart patterning and the induction of *tin*-dependent muscle founder cells in the dorsal mesoderm [12], [34]. The *eve* MHE enhancer tested for Pnt binding does contain CEE motifs, although they were not included in the mutational analysis by Halfon et al. [12]. The CEE motif also has some resemblance to activating half sites of the NFκB factor Dorsal [60], [61], but Dorsal binds to half sites only poorly, if at all [62], and is present solely in the cytoplasm with undetectable nuclear levels at the stages when the CEE-dependent enhancers become active (A. O., unpublished data). For these reasons, we currently do not favor Dorsal as an essential CEE-binding trans-activator. Additional studies, including the determination of the regions bound by Pnt and Dorsal *in vivo* during the relevant stages, and genetic as well as biochemical assays, are needed to clarify whether either Pnt or Dorsal might correspond to the presumed CEE motif binding factor or whether another factor is involved. In the near future, the availability of an increasing number of enhancers active in the cardiac mesoderm and other mesodermal tissues will greatly improve the reliability of machine learning approaches to detect novel functional motifs in tissue-specific enhancers.

## Materials and Methods

### ChIP–chip

Embryo crosslinking, chromatin isolation, chromatin immunoprecipitation and DNA amplification were carried out according to the protocol described previously in detail [38], except that Protein A/G beads (Pierce) were used for the immunoprecipitation. Wild-type embryo populations were collected at 3–5.5 and 5–8 hrs after egg laying, respectively. For each developmental time period, two independent ChIP experiments were performed. The polyclonal anti-Tin antibody was obtained by affinity purification with recombinant Tin from rabbit anti-Tin serum [4]. Normal rabbit IgG was used as the mock control. The final amplified and labeled DNA samples were hybridized to GeneChip *Drosophila* Tiling 1.0R Arrays (Affymetrix) at the microarray core facility of Mount Sinai School of Medicine (New York, NY). ChIP peaks were called using Model-based Analysis of Tiling-arrays (MAT) [63] using a default P-value threshold of 1e-5. The accession number for the dataset in the Gene Expression Omnibus (GEO) database is GSE41628. The data were mapped to the UCSC dm3 genome assembly reference genome.

### *Drosophila* strains

The strains *M{eGFP.vas-int.Dm}ZH-2A;M{RFP.attP}ZH-51C* and *M{eGFP.vas-int.Dm}ZH-2A;M{RFP.attP}ZH-35B* [64] were used for germline transformation of the reporter constructs. The strains *tin^346^/TM3,eve-lacZ* and *tin-ABD;tin^346^/TM3,eve-lacZ* [9] were used for analyzing the Tin dependency of enhancer activities.

### Generation of reporter constructs

Genomic fragments with the coordinates shown in Tables S4 and S5 were amplified from genomic DNA of Oregon R by PCR, and cloned into either the pH-Stinger-attB vector, which was constructed by inserting the attB sequence [65] into the AvrII site of the pH-Stinger vector, and injected into *ZH-35B* (at Erlangen or Duke University Model System Genomics, Durham, NC). For AlkE301, AopE53, AopL18, CG5522E11, EgfrE1, EgfrE63, gukhE135, meso18EE403, mipleE8, NrtL30, nvyE164, slpE35, tshL8 and wgnE1197, cloning was done into the eve.promoter-LacZ-attB vector [66] and injections into *ZH-51C* (at Rainbow Transgenic Flies, Inc., Camarillo, CA). Although located closer to the genes *Her* and *Dot*, respectively, the enhancer activities of HimL47 and oddE81 clearly reflected specific aspects of the neighboring genes *Him* and *odd* and were named accordingly. The Tin and Doc sites within the genomic fragments were predicted using the web-based tool Target Explorer [67]. Alignment scores >4.5 using the same score matrix for Tin as for Tin motif shown in Figure 1 (see also Table S5) were given higher priority for selection of candidate enhancer fragments. Mutations of these motifs and the GATA sequences were obtained via *de novo* DNA synthesis (Mr. Gene, Regensburg, DE; Biomatik, Cambridge ON, CA) (Table S6). For Target Explorer, the position weight matrix for Tin was built with Tin-binding sites verified experimentally and *in vivo* (Table S5) [4], [6], [18], [22], [68]–[70]. Construction of the position weight matrices for Doc2 and Mid was based on the results of SELEX experiments with recombinant Doc2_T-box-GST and Mid_T-box-GST, which were performed following a GST pull-down version of the SELEX method described previously (Table S5) [71].

### *In situ* hybridization

Whole mount embryo *in situ* hybridization was carried out following standard protocol, as described previously [69]. The digoxigenin-labeled probe targeting the eGFP transcript was synthesized by *in vitro* transcription of eGFP sequence inserted into the TOPO cloning site of the pCRII-TOPO vector.

### Immunostaining

Immunostaining of embryos using DAB and double or triple fluorescent immunostaining were carried out as described previously [69]. The following primary antibodies were used: rabbit anti-GFP (1∶2000, Invitrogen), rabbit anti-β-gal (1∶1200, Promega), rabbit anti-Tin (1∶1000, this study), mouse anti-GFP monoclonal (1∶1000, Invitrogen), mouse anti-β-gal monoclonal (1∶50, Developmental Studies Hybridoma Bank), guinea pig anti-Doc2/3 (1∶400) [72], rabbit anti-Eve (1∶3000) [73]. For fluorescent detection, FITC-, Cy3-, or DyLight-conjugated secondary antibodies (Jackson Laboratories) were used. For monoclonal primary antibodies, Tyramide Signal Amplification (TSA) was performed using biotinylated secondary antibodies (1∶500, Vector Laboratories) in combination with the Vectastain ABC Kit (Vector Laboratories) and fluorescent Tyramide Reagent (PerkinElmer). The images of stainings were acquired using the Zeiss ApoTome/AxioImager.Z1 microscope system with Plan-Apochromat 20×/0,8.

### *De novo* motif discovery

We used RSAT tools oligo-analysis and oligo-diff to find *de novo* motifs [32]. For both Early and Late datasets we used oligo-analysis with default parameters on the top 100 peaks. On the datasets of all peaks we found that with default parameters the list of *de novo* motifs is dominated with repeats, thus we use Markov background model of 3rd order and performed Repeat Masking [33]. We used default parameters for oligo-analysis and oligo-diff to discover motifs in subset of experimentally verified enhancers. We ran RSAT on peak regions defined by MAT, but also on the full experimentally verified fragments with and without Repeat Masking. The resulting *de novo* motifs were curated by hand to remove duplicates and merge similar motifs (consensus sequences different in one nucleotide). The CEE motif was derived by merging a consensus motif enriched in Repeat Masked cardiac enhancers and a consensus motif discovered by oligo-diff when comparing cardiac with somatic enhancers. The somatic1 motif (Figure 6) was discovered as being enriched in somatic enhancers compared to cardiac using oligo-diff.

### Machine learning

We used a classification approach similar to [74]. The set of 61 enhancers was scanned with a database of motifs we derived from: JASPAR insects collection [75], ChIP-chip for key mesodermal TFs [25], Dan Pollard's MEME motifs from FlyReg [76] and *de novo* motifs (as described above). We used a log-odds threshold of 4.6 to call significant motif hits with a Markov background of order 2. A LASSO classifier was trained to predict cardiac enhancers from the set of 61 experimentally verified enhancers based on the density of motif hits (defined as number of motif hits per kb of sequence). Motif hit density was used because the sequences were of different lengths. The classifier was trained on 1000 bootstrap samples of 61 enhancers and the regularization parameter picked so to minimize cross-validation error with the maximal of 50 features selected. We reasoned that if a classifier picked more than 50 features it would most certainly be over-fitting the data. The final classifier (Figure 6) was constructed by taking all features that were present in at least 65% of bootstrap runs. We picked this threshold by starting with 95% and then decreasing it as long as the AUC-ROC of the classifier improved. This yielded a classifier with Area Under the ROC Curve (AUC-ROC) score of 0.90 obtained from Leave-one-out cross validation (LOOCV).

### Functional analysis of target genes

We assigned each ChIP peak to the nearest gene boundary (5′ or 3′ exon) from the peak maximal enrichment point in either direction because a number of key mesodermal enhancers are known to be downstream of their respective target genes. If the peak was in an intron, we assigned it to the nearest transcription start site of the nested gene. We performed GO enrichment analysis using Ontologizer [77] using the Topology-weight algorithm to account for the hierarchical structure of GO ontologies [78]. We used FlyMine to perform protein domain enrichment analysis [79]. We used the BDGP *in situ* database release 3 [28] and we only retained those entries that were marked of “acceptable” quality. We performed enrichment analysis using a standard hypergeometic test with False Discovery Rate (FDR) correction.

### Motif location analysis

We analysed motif location (Figure 1) by taking all defined ChIP regions and scanning them with the appropriate *de novo* motifs. Significant motif hits were called above log-odds threshold of 4.5 and their position recorded in percentages relative to peak region width. Thus, we used the original peak positions without trimming them to a fixed size. The motif counts were then binned in 5% bins and the resulting histogram smoothed using loess smoothing. We scaled the density so that number 1 represents the expected uniform distribution. By scanning with unrelated PWMs we verified that the expected distribution is indeed a flat line at 1 (data not shown).

## Supporting Information

Figure S1 Analysis of peak locations and peak overlaps with other studies. (A) Peak positions in Tin Early dataset. We assigned each peak to the target gene as described in Materials and Methods. A great majority of the peaks fall into introns and intergenic regions as expected and only a small number into exons. (B) Peak positions in Tin Late dataset. Although the Late dataset is considerably smaller, the peak distribution over regions of the genome is very similar. (C) Venn diagrams of Tin Early and Late overlap with those from ref. [25]. We overlapped Tin Early and Late dataset with Zinzen et al. Tin 4–6 h and Tin 6–8 h datasets. Since the peaks do not always map one-to-one (e.g., one dataset might call two smaller peaks instead of one larger), the numbers in overlaps correspond to Tin Early peaks, except for the overlaps between the Tin Late and Tin 4–6/Tin 6–8 h datasets where the numbers correspond to Tin Late peaks. The numbers outside the overlaps are of remaining peaks in each of the dataset. The Tin 4–6 h dataset is more similar to Tin Early (3–5.5 h), while Tin 6–8 h dataset is more similar to Tin Late (5–8 h). Furthermore, there is a core set of 394 overlapping peaks (not shown in the Figure) between all four datasets. (D) Table of full Tin Early and Late overlaps with those from ref. [25] and modENCODE HOT regions with complexity over 8 [27]. Percentages are relative to the number of peaks of the dataset in table row. Both the Tin Early and Tin Late dataset, and also the Tin datasets from ref. [25], show significant overlaps of 36–46% with the HOT regions.
(TIF)

Figure S2 Activity patterns of Tin-bound enhancers in somatic mesoderm and muscles. Genes are ordered alphabetically, with CGs last. (A, A′) Expression of *commE28*-GFP in lateral somatic mesoderm (A, stage 11) and lateral muscle precursors (A′, stage 14). (B, B′) Expression of *fendE160*-GFP in all somatic mesoderm (B, sm, stage 13) and somatic muscles (B′, sm, stage 15), as well as in lateral ectoderm (ec). (C, C′) Expression of *H15L5*-GFP in somatic muscle precursors (C, stage 14) and muscles, as well as in amnioserosa (C′, stage 15). (D, D′) Expression of *hhL2*-GFP in somatic mesoderm (D, stage 10) and muscles ((D′, stage 15). (E, E′) Expression of *lbeL170*-GFP in lateral somatic mesodermal cell clusters encompassing the area around the SBM founder cells and the lateral adult muscle precursors (E, stage 11; E′, stage 13). (F, F′) Striped expression of *meso18EE403*-LacZ in somatic mesoderm (F, sm, stage 10), residual expression in the somatic mesoderm at stage 14 (F′, sm), and robust expression in the anterior visceral mesoderm at stage 14 (F′, vm). (G, G′) Striped expression of *PoxmE138*-GFP in the ventral somatic mesoderm (G, stage 11, ventral view) and in ventral somatic muscles, especially muscles 26, 27, 29 (G′, stage 16). (H, H′) Expression of *PscE14*-GFP in somatic mesoderm (H, stage 11) and somatic muscles (H′, stage 16). (I, I′) Expression of *Six4E255*-GFP in ventral and lateral somatic mesoderm (I, stage 11) and ventral/lateral muscle precursors (I′, stage 14). (J, J′) Expression of *slpE35*-GFP in segmental stripes in somatic mesoderm at stage 10 (J, optical section) and stage 11 (J′, on-view). (K, K′) Expression of *zfh1E141*-GFP in somatic mesoderm (K, stage 10) and muscle precursors (K′, stage 13). (L, L′) Expression of *CG5522E11*-GFP in somatic mesoderm and ectodermal stripes (L, stage 10, lateral view; L′, stage 11, ventral view). (M, M′) Expression of *CG10479L38*-GFP in somatic muscle precursors (sm), salivary glands, and endoderm (M, stage 13, ventral view; M′, stage 14, lateral view). (N, N′) Striped expression of *CG32792E165*-GFP in somatic mesoderm (N, stage 11) and in somatic muscle precursors (N′, stage 14).
(TIF)

Figure S3 Activity patterns of Tin-bound enhancers in trunk visceral mesoderm and fat body precursors. (A, A′) Expression of *AlkE301*-LacZ in visceral mesoderm precursors (A, stage 10, ventral view) and visceral mesoderm (A′, stage 12, lateral view). (B, B′) Expression of *Fas3L254*-GFP in visceral mesoderm precursors (B, stage 10) and visceral mesoderm (B′, stage 14). (C, C′) Expression of *gukhE135*-LacZ in visceral mesoderm (C, stage 12; C′, stage 15; arrows), as well as in epidermal cells. (D, D′) Expression of *H2.0L95*-GFP in trunk visceral muscle founders and caudal visceral mesoderm (D, stage 12) and in trunk visceral mesoderm and longitudinal visceral muscle precursors (D′, stage 14). (E, E′) Expression of *NrtL30*-LacZ in trunk visceral muscle founders and epidermal stripes (E, stage 11, ventral view), and in visceral musculature (E′, stage 15). (F, F′) Expression of wgnE1197-LacZ in mesodermal pair-rule stripes (F, stage 9), and in visceral musculature of proventriculus and posterior midgut (F′, stage 16). (G, G′) Expression of *oddE81*-GFP in fat body primordia (F, stge 10) and developing lateral fat body (F′, stage 14), an expression that reflects one aspect of the endogenous *odd* mRNA pattern.
(TIF)

Figure S4 Activity patterns of Tin-bound enhancers in *tin*-dependent dorsal median cells. (A, A′) Expression of *btnE455*-GFP in dorsal median cells (A, stage 11, lateral view; A′, stage 14, ventral view). (B, B′) Expression of *Ten-mE146*-GFP in dorsal median cells and developing head CNS (B, stage 11; B′, stage 15).
(TIF)

Figure S5 Activity patterns of Tin-bound enhancers in non-mesodermal tissues. (A, A′) Expression of *abd-AL104*-GFP in striped ventral ectodermal domains (A, stage 10) and in the stomatogastric nervous system (A′, stage 16). (B, B′) Expression of *AntpL15*-GFP in epidermal areas of the maxillary segment and in single ectodermally-derived clusters of cells in each thoracic segment (B, stage 14). At stage 15 (B′), *AntpL15*-GFP is present in head areas, maxilla, and the ring gland. (C, C′) Expression of *AopE53*-LacZ at stage 13 in most epidermal cells (C, optical section; C′, superficial view). (D, D′) Expression of *AopL18*-LacZ at stage 13 (D) and stage 14 (D′) in epidermal cells of gnathal segments and in ventrolateral segmental areas of thoracic and abdominal segments. (E, E′) Expression of *caupL37*-GFP in the stomatogastric nervous system (E, stage 15; E′, stage 16). (F, F′) Expression of *commE137*-GFP largely in lateral, segmental clusters of ectodermally derived cells (presumably specific sense organ progenitors; F, stage 12; F′, stage 14). (G, G′) Expression of *DiscoL286*-GFP in endodermal cells (presumably corresponding to *labial*-positive copper cells; G, stage 14; G′, stage 15). (H, H′) Expression of *DrE59*-GFP in the lateral column of neuroectodermal cells (H, stage 10) and in foregut, hindgut, and salivary gland (H′, stage 15). (I, I′) Expression of *EgfrE63*-LacZ in lateral ectodermal cells (I, stage 10) and the roof of the stomodeum (I′, stage 14). (J, J′) Expression of *mipleE8*-LacZ in the endoderm (J, stage 12) and in anterior head tissues (J′, stage 16). (K, K′) Expression of *nvyE164*-LacZ in peripheral nervous system progenitors (K, stage 11) and PNS (K′, stage 15). (L, L′) Uniform epidermal expression of *shgE33*-GFP (L, stage 11; L′, stage 14).
(TIF)

Figure S6 Dependence of the activities of selected Tin-bound enhancers on *tin*. Shown are stage 11–12 wild type embryos (A–K) and homozygous *tin^346^* embryos (A′–K′) carrying the denoted *lacZ* or *GFP* reporter constructs and stained with anti-β-Gal or anti-GFP (red) and anti-Tin (green). (A) Tin protein and *AlkE301*-LacZ co-localize in trunk visceral mesoderm. (A′) In the absence of *tin* activity, *AlkE301*-lacZ is no longer expressed in the trunk visceral mesoderm. (B) Expression of *btnE455*-GFP in dorsal median cells after Tin has become restricted to dorsal mesoderm. (B′) Lack of *btnE455*-GFP expression at the normal positions of dorsal median cells. (C) Co-expression of *Fas3L254*-GFP with Tin in the trunk visceral mesoderm. (C′) Severe reduction of *Fas3 L254*-GFP expression in the absence of *tin* activity. (D) Co-expression of *hthE54*-GFP with Tin in anterior cardiogenic mesoderm (arrows). (D′) Lack of *hthE54*-GFP expression in anterior cardiogenic mesoderm (arrows). Somatic mesodermal *hthE54*-GFP is *tin*-independent. (E) Expression of *lbeL170*-GFP in somatic mesodermal clusters after Tin has been restricted to dorsal mesoderm. (E′) Near lack of *lbeL170*-GFP expression in the absence of *tin* activity. (F) Co-expression of *nauL35*-GFP and Tin in dorsal somatic mesoderm clusters. (F′) Lack of *nauL35*-GFP expression in dorsal somatic mesoderm in the absence of *tin* activity. (G) Co-expression of *nocL7*-GFP and Tin in cardiogenic mesoderm, and segmented expression in dorsal somatic mesoderm. (G′) Both cardiogenic and somatic mesodermal *nocL7*-GFP depend on *tin* activity. (H) Co-expression of *NrtL30*-LacZ and Tin in the trunk visceral mesoderm (arrows). (H′) Lack of *NrtL30*-LacZ activity in trunk visceral mesoderm in the absence of *tin* activity. Ectodermal GFP is unaffected. (I) *Six4E255*-GFP activity in segmental stripes within the ventrolateral somatic mesoderm after Tin has been restricted to the dorsal mesoderm. (I′) Slight reduction of *Six4E255*-GFP expression levels in the absence of *tin*. (J) *zfh1E141*-GFP expression in entire mesoderm at the time when Tin has become restricted to the cardiogenic and parts of the visceral mesoderm. (J′) In the absence of *tin* activity there is no significant change in *zfh1E141*-GFP activity.
(TIF)

Table S1 Summary of Tin peaks and their gene assignments in the Tinman Early and Late datasets.
(XLS)

Table S2 GO terms and protein domains enriched in genes associated with Tin binding peaks. A) GO terms. B) Protein domains.
(XLS)

Table S3 Enrichment of BDGP *in situ* hybridization terms. A) Terms related to Tin expression domains. B) All terms.
(PDF)

Table S4 Integrated tables of Tin binding peaks from 3–5.5 h (top 455 peaks) and 5–8 h (top 443 peaks) datasets. A) 3–5.5 h dataset. B) 5–8 h dataset.
(XLS)

Table S5 List of fragments tested *in vivo* and in machine learning approach. Summary of all enhancer data, binding motifs present, and comparison of the *in vivo* occupancy of these enhancer regions by various factors tested in Junion et al. (2012) [26].
(XLS)

Table S6 List of mutated sequences.
(XLS)
