# Supplementary figures and images for: Genome-Wide Screens for In Vivo Tinman Binding Sites Identify Cardiac Enhancers with Diverse Functional Architectures
Source: PLoS Genet. 2013 Jan 10;9(1):e1003195. doi: 10.1371/journal.pgen.1003195 (PMC3542182; doi:10.1371/journal.pgen.1003195)

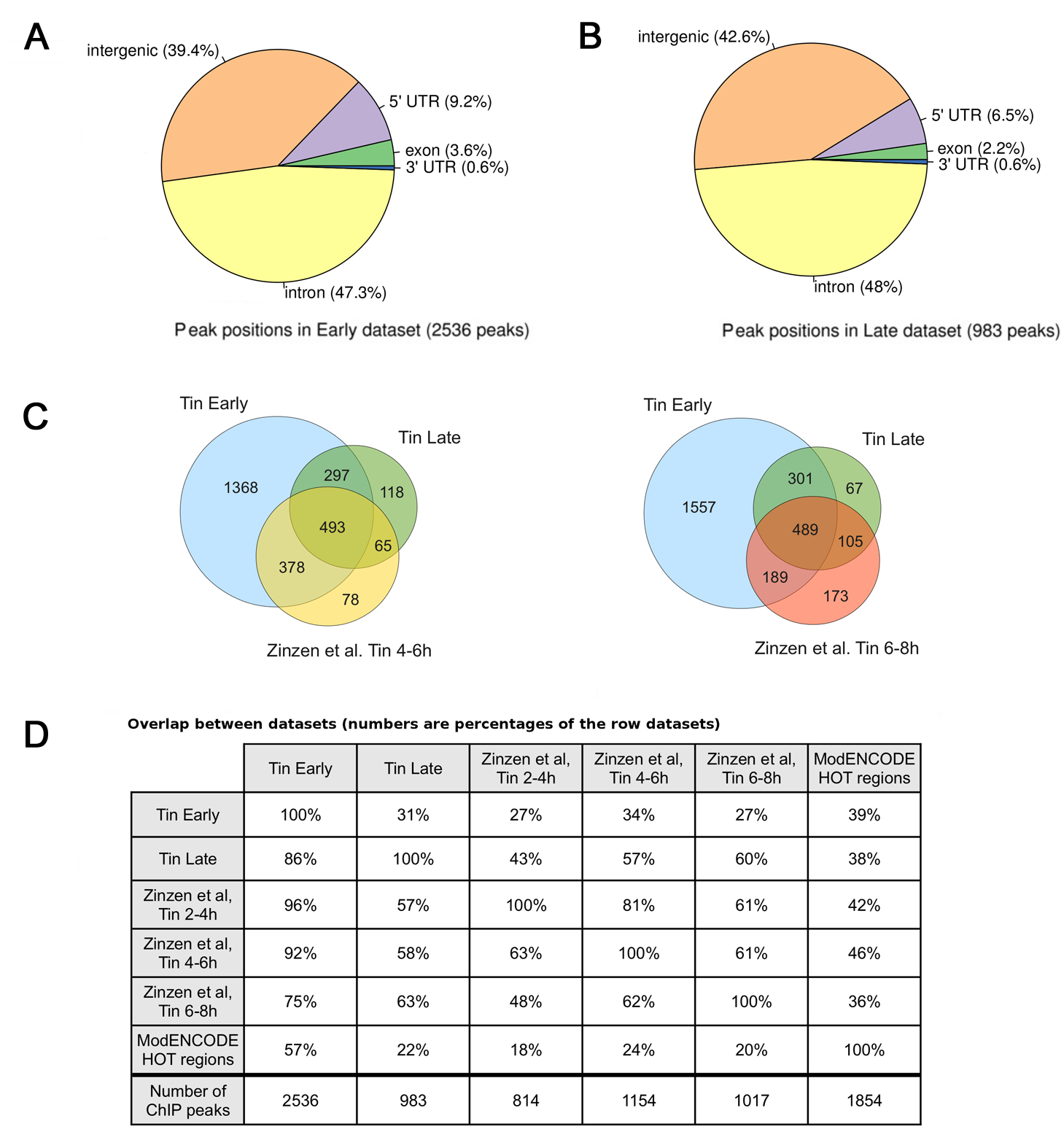

Supplement: Figure S1 — Analysis of peak locations and peak overlaps with other studies. (A) Peak positions in Tin Early dataset. We assigned each peak to the target gene as described in Materials and Methods. A great majority of the peaks fall into introns and intergenic regions as expected and only a small number into exons. (B) Peak positions in Tin Late dataset. Although the Late dataset is considerably smaller, the peak distribution over regions of the genome is very similar. (C) Venn diagrams of Tin Early and Late overlap with those from ref. [25]. We overlapped Tin Early and Late dataset with Zinzen et al. Tin 4–6 h and Tin 6–8 h datasets. Since the peaks do not always map one-to-one (e.g., one dataset might call two smaller peaks instead of one larger), the numbers in overlaps correspond to Tin Early peaks, except for the overlaps between the Tin Late and Tin 4–6/Tin 6–8 h datasets where the numbers correspond to Tin Late peaks. The numbers outside the overlaps are of remaining peaks in each of the dataset. The Tin 4–6 h dataset is more similar to Tin Early (3–5.5 h), while Tin 6–8 h dataset is more similar to Tin Late (5–8 h). Furthermore, there is a core set of 394 overlapping peaks (not shown in the Figure) between all four datasets. (D) Table of full Tin Early and Late overlaps with those from ref. [25] and modENCODE HOT regions with complexity over 8 [27]. Percentages are relative to the number of peaks of the dataset in table row. Both the Tin Early and Tin Late dataset, and also the Tin datasets from ref. [25], show significant overlaps of 36–46% with the HOT regions. (TIF) [file pgen.1003195.s001.tif]

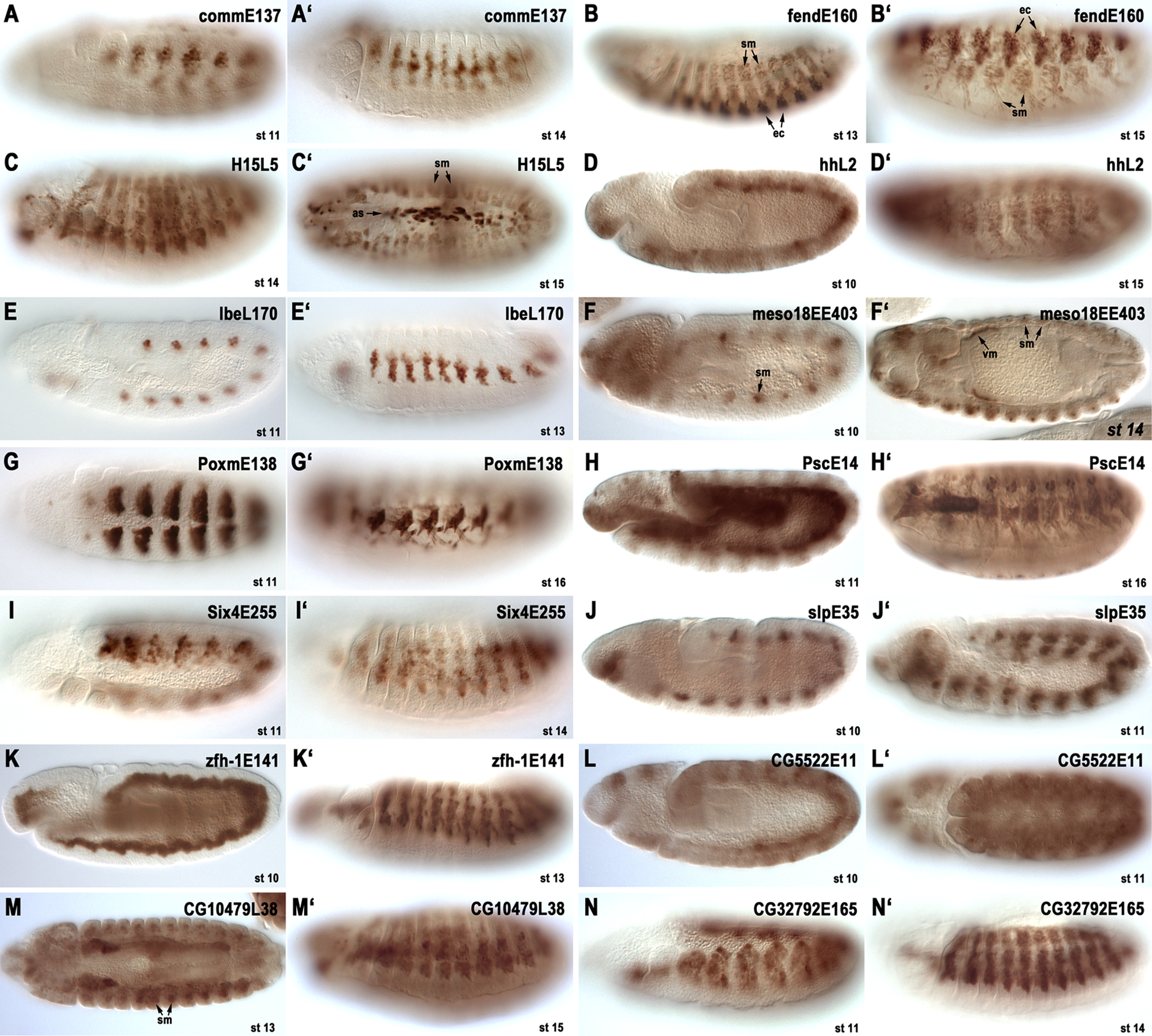

Supplement: Figure S2 — Activity patterns of Tin-bound enhancers in somatic mesoderm and muscles. Genes are ordered alphabetically, with CGs last. (A, A′) Expression of commE28-GFP in lateral somatic mesoderm (A, stage 11) and lateral muscle precursors (A′, stage 14). (B, B′) Expression of fendE160-GFP in all somatic mesoderm (B, sm, stage 13) and somatic muscles (B′, sm, stage 15), as well as in lateral ectoderm (ec). (C, C′) Expression of H15L5-GFP in somatic muscle precursors (C, stage 14) and muscles, as well as in amnioserosa (C′, stage 15). (D, D′) Expression of hhL2-GFP in somatic mesoderm (D, stage 10) and muscles ((D′, stage 15). (E, E′) Expression of lbeL170-GFP in lateral somatic mesodermal cell clusters encompassing the area around the SBM founder cells and the lateral adult muscle precursors (E, stage 11; E′, stage 13). (F, F′) Striped expression of meso18EE403-LacZ in somatic mesoderm (F, sm, stage 10), residual expression in the somatic mesoderm at stage 14 (F′, sm), and robust expression in the anterior visceral mesoderm at stage 14 (F′, vm). (G, G′) Striped expression of PoxmE138-GFP in the ventral somatic mesoderm (G, stage 11, ventral view) and in ventral somatic muscles, especially muscles 26, 27, 29 (G′, stage 16). (H, H′) Expression of PscE14-GFP in somatic mesoderm (H, stage 11) and somatic muscles (H′, stage 16). (I, I′) Expression of Six4E255-GFP in ventral and lateral somatic mesoderm (I, stage 11) and ventral/lateral muscle precursors (I′, stage 14). (J, J′) Expression of slpE35-GFP in segmental stripes in somatic mesoderm at stage 10 (J, optical section) and stage 11 (J′, on-view). (K, K′) Expression of zfh1E141-GFP in somatic mesoderm (K, stage 10) and muscle precursors (K′, stage 13). (L, L′) Expression of CG5522E11-GFP in somatic mesoderm and ectodermal stripes (L, stage 10, lateral view; L′, stage 11, ventral view). (M, M′) Expression of CG10479L38-GFP in somatic muscle precursors (sm), salivary glands, and endoderm (M, stage 13, ventral view; M′, stage 14, [file pgen.1003195.s002.tif]

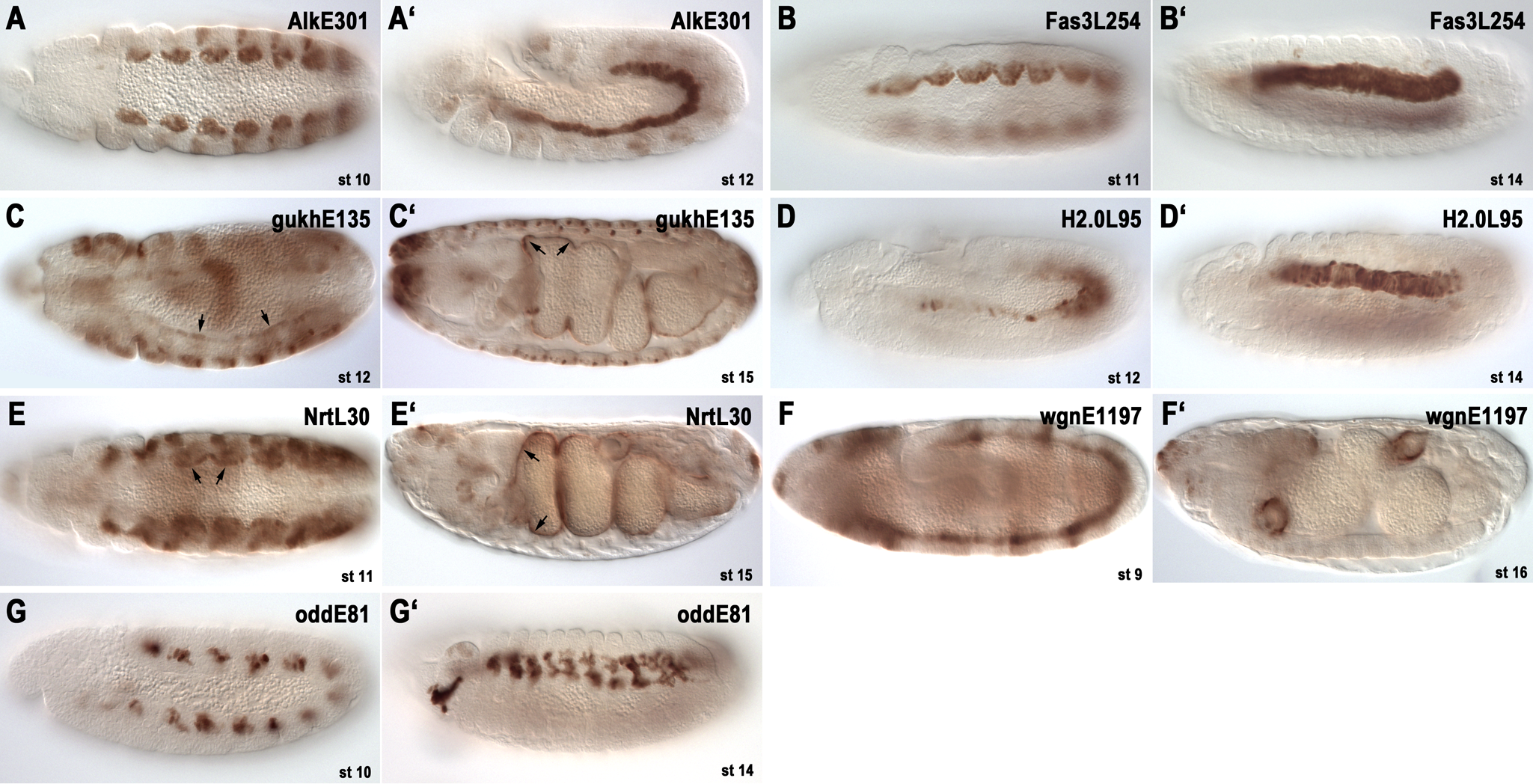

Supplement: Figure S3 — Activity patterns of Tin-bound enhancers in trunk visceral mesoderm and fat body precursors. (A, A′) Expression of AlkE301-LacZ in visceral mesoderm precursors (A, stage 10, ventral view) and visceral mesoderm (A′, stage 12, lateral view). (B, B′) Expression of Fas3L254-GFP in visceral mesoderm precursors (B, stage 10) and visceral mesoderm (B′, stage 14). (C, C′) Expression of gukhE135-LacZ in visceral mesoderm (C, stage 12; C′, stage 15; arrows), as well as in epidermal cells. (D, D′) Expression of H2.0L95-GFP in trunk visceral muscle founders and caudal visceral mesoderm (D, stage 12) and in trunk visceral mesoderm and longitudinal visceral muscle precursors (D′, stage 14). (E, E′) Expression of NrtL30-LacZ in trunk visceral muscle founders and epidermal stripes (E, stage 11, ventral view), and in visceral musculature (E′, stage 15). (F, F′) Expression of wgnE1197-LacZ in mesodermal pair-rule stripes (F, stage 9), and in visceral musculature of proventriculus and posterior midgut (F′, stage 16). (G, G′) Expression of oddE81-GFP in fat body primordia (F, stge 10) and developing lateral fat body (F′, stage 14), an expression that reflects one aspect of the endogenous odd mRNA pattern. (TIF) [file pgen.1003195.s003.tif]

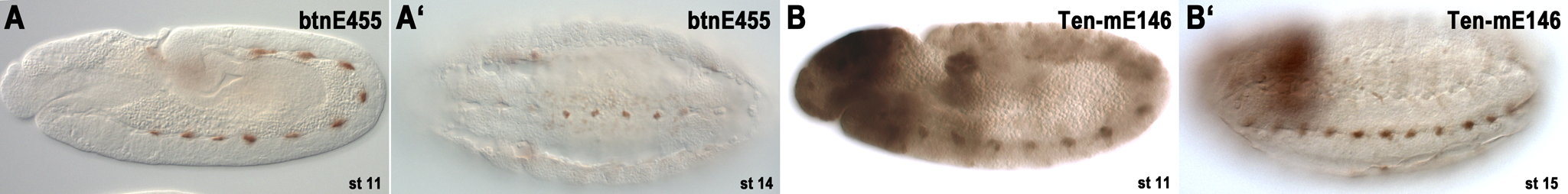

Supplement: Figure S4 — Activity patterns of Tin-bound enhancers in tin-dependent dorsal median cells. (A, A′) Expression of btnE455-GFP in dorsal median cells (A, stage 11, lateral view; A′, stage 14, ventral view). (B, B′) Expression of Ten-mE146-GFP in dorsal median cells and developing head CNS (B, stage 11; B′, stage 15). (TIF) [file pgen.1003195.s004.tif]

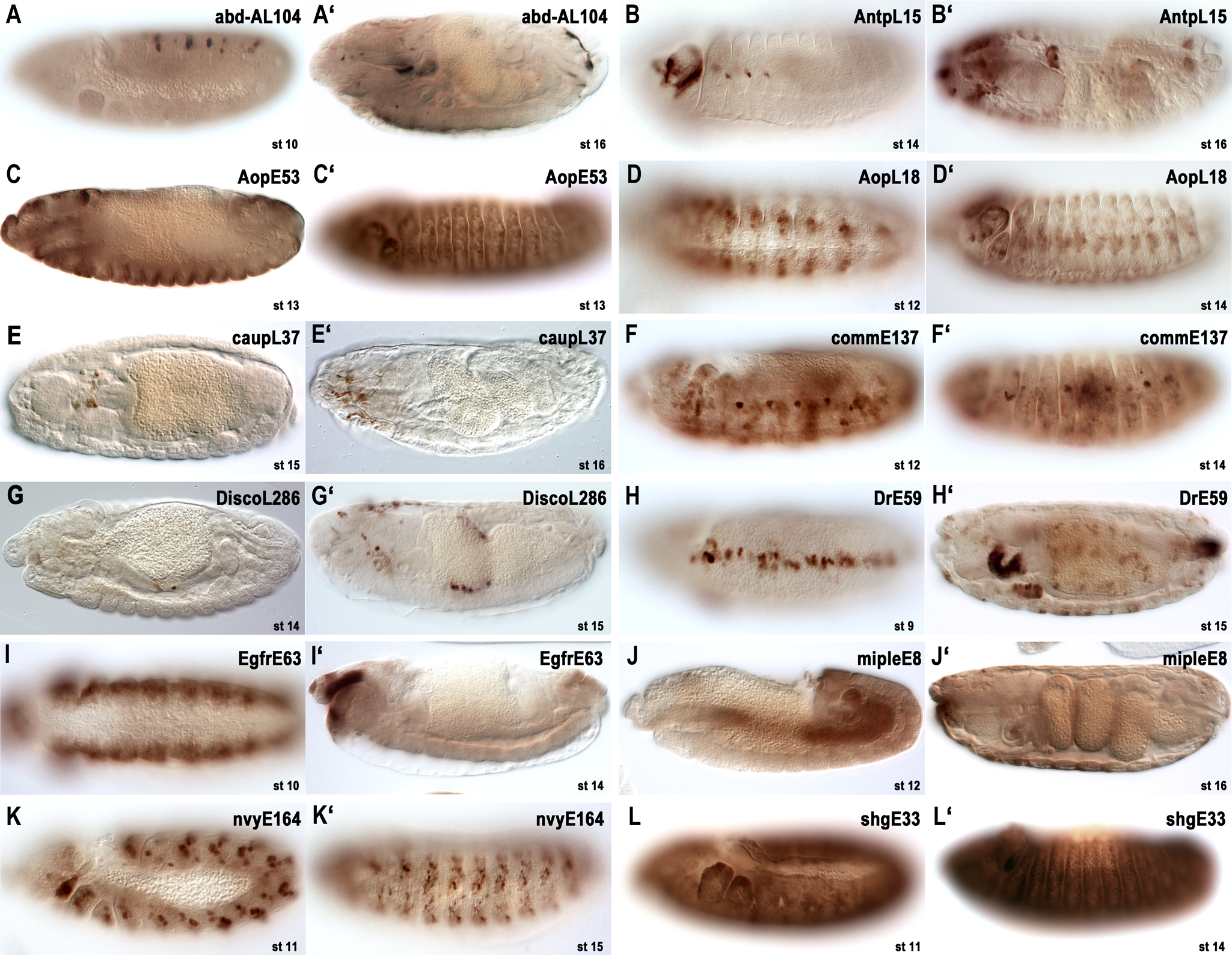

Supplement: Figure S5 — Activity patterns of Tin-bound enhancers in non-mesodermal tissues. (A, A′) Expression of abd-AL104-GFP in striped ventral ectodermal domains (A, stage 10) and in the stomatogastric nervous system (A′, stage 16). (B, B′) Expression of AntpL15-GFP in epidermal areas of the maxillary segment and in single ectodermally-derived clusters of cells in each thoracic segment (B, stage 14). At stage 15 (B′), AntpL15-GFP is present in head areas, maxilla, and the ring gland. (C, C′) Expression of AopE53-LacZ at stage 13 in most epidermal cells (C, optical section; C′, superficial view). (D, D′) Expression of AopL18-LacZ at stage 13 (D) and stage 14 (D′) in epidermal cells of gnathal segments and in ventrolateral segmental areas of thoracic and abdominal segments. (E, E′) Expression of caupL37-GFP in the stomatogastric nervous system (E, stage 15; E′, stage 16). (F, F′) Expression of commE137-GFP largely in lateral, segmental clusters of ectodermally derived cells (presumably specific sense organ progenitors; F, stage 12; F′, stage 14). (G, G′) Expression of DiscoL286-GFP in endodermal cells (presumably corresponding to labial-positive copper cells; G, stage 14; G′, stage 15). (H, H′) Expression of DrE59-GFP in the lateral column of neuroectodermal cells (H, stage 10) and in foregut, hindgut, and salivary gland (H′, stage 15). (I, I′) Expression of EgfrE63-LacZ in lateral ectodermal cells (I, stage 10) and the roof of the stomodeum (I′, stage 14). (J, J′) Expression of mipleE8-LacZ in the endoderm (J, stage 12) and in anterior head tissues (J′, stage 16). (K, K′) Expression of nvyE164-LacZ in peripheral nervous system progenitors (K, stage 11) and PNS (K′, stage 15). (L, L′) Uniform epidermal expression of shgE33-GFP (L, stage 11; L′, stage 14). (TIF) [file pgen.1003195.s005.tif]

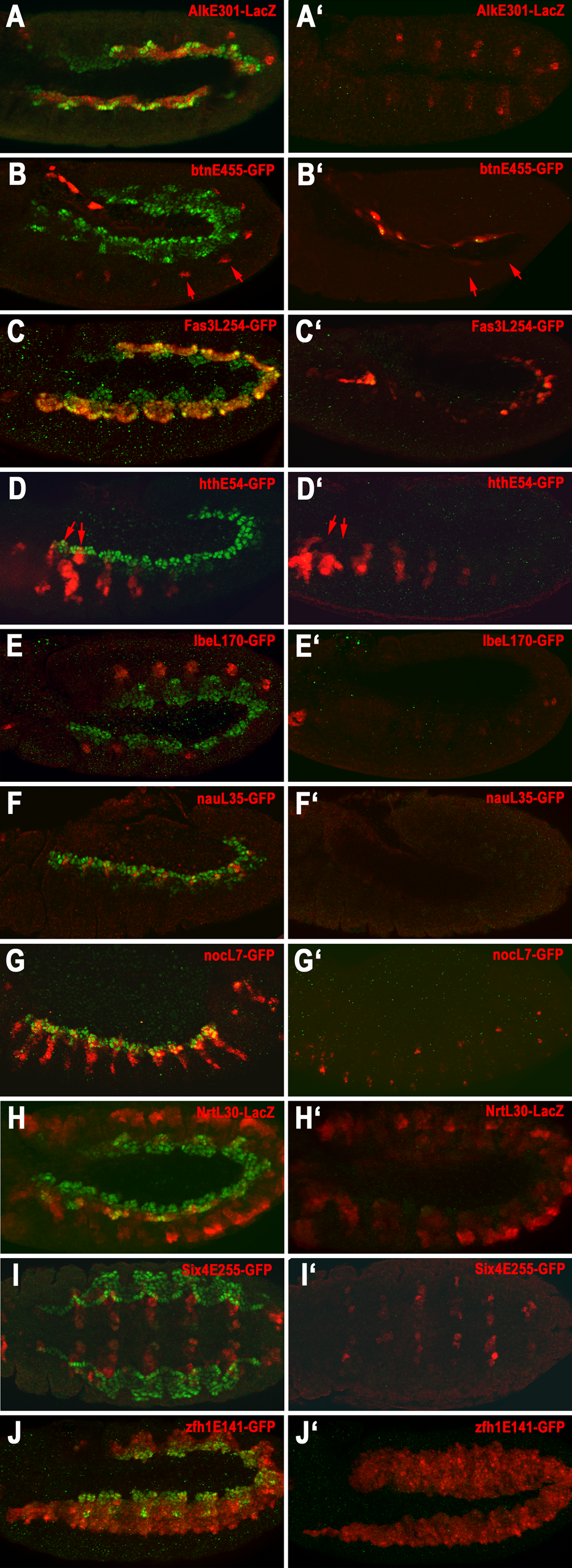

Supplement: Figure S6 — Dependence of the activities of selected Tin-bound enhancers on tin. Shown are stage 11–12 wild type embryos (A–K) and homozygous tin346 embryos (A′–K′) carrying the denoted lacZ or GFP reporter constructs and stained with anti-β-Gal or anti-GFP (red) and anti-Tin (green). (A) Tin protein and AlkE301-LacZ co-localize in trunk visceral mesoderm. (A′) In the absence of tin activity, AlkE301-lacZ is no longer expressed in the trunk visceral mesoderm. (B) Expression of btnE455-GFP in dorsal median cells after Tin has become restricted to dorsal mesoderm. (B′) Lack of btnE455-GFP expression at the normal positions of dorsal median cells. (C) Co-expression of Fas3L254-GFP with Tin in the trunk visceral mesoderm. (C′) Severe reduction of Fas3 L254-GFP expression in the absence of tin activity. (D) Co-expression of hthE54-GFP with Tin in anterior cardiogenic mesoderm (arrows). (D′) Lack of hthE54-GFP expression in anterior cardiogenic mesoderm (arrows). Somatic mesodermal hthE54-GFP is tin-independent. (E) Expression of lbeL170-GFP in somatic mesodermal clusters after Tin has been restricted to dorsal mesoderm. (E′) Near lack of lbeL170-GFP expression in the absence of tin activity. (F) Co-expression of nauL35-GFP and Tin in dorsal somatic mesoderm clusters. (F′) Lack of nauL35-GFP expression in dorsal somatic mesoderm in the absence of tin activity. (G) Co-expression of nocL7-GFP and Tin in cardiogenic mesoderm, and segmented expression in dorsal somatic mesoderm. (G′) Both cardiogenic and somatic mesodermal nocL7-GFP depend on tin activity. (H) Co-expression of NrtL30-LacZ and Tin in the trunk visceral mesoderm (arrows). (H′) Lack of NrtL30-LacZ activity in trunk visceral mesoderm in the absence of tin activity. Ectodermal GFP is unaffected. (I) Six4E255-GFP activity in segmental stripes within the ventrolateral somatic mesoderm after Tin has been restricted to the dorsal mesoderm. (I′) Slight reduction of Six4E255-GFP expression levels in the absence of tin. (J) zfh1E141 [file pgen.1003195.s006.tif]

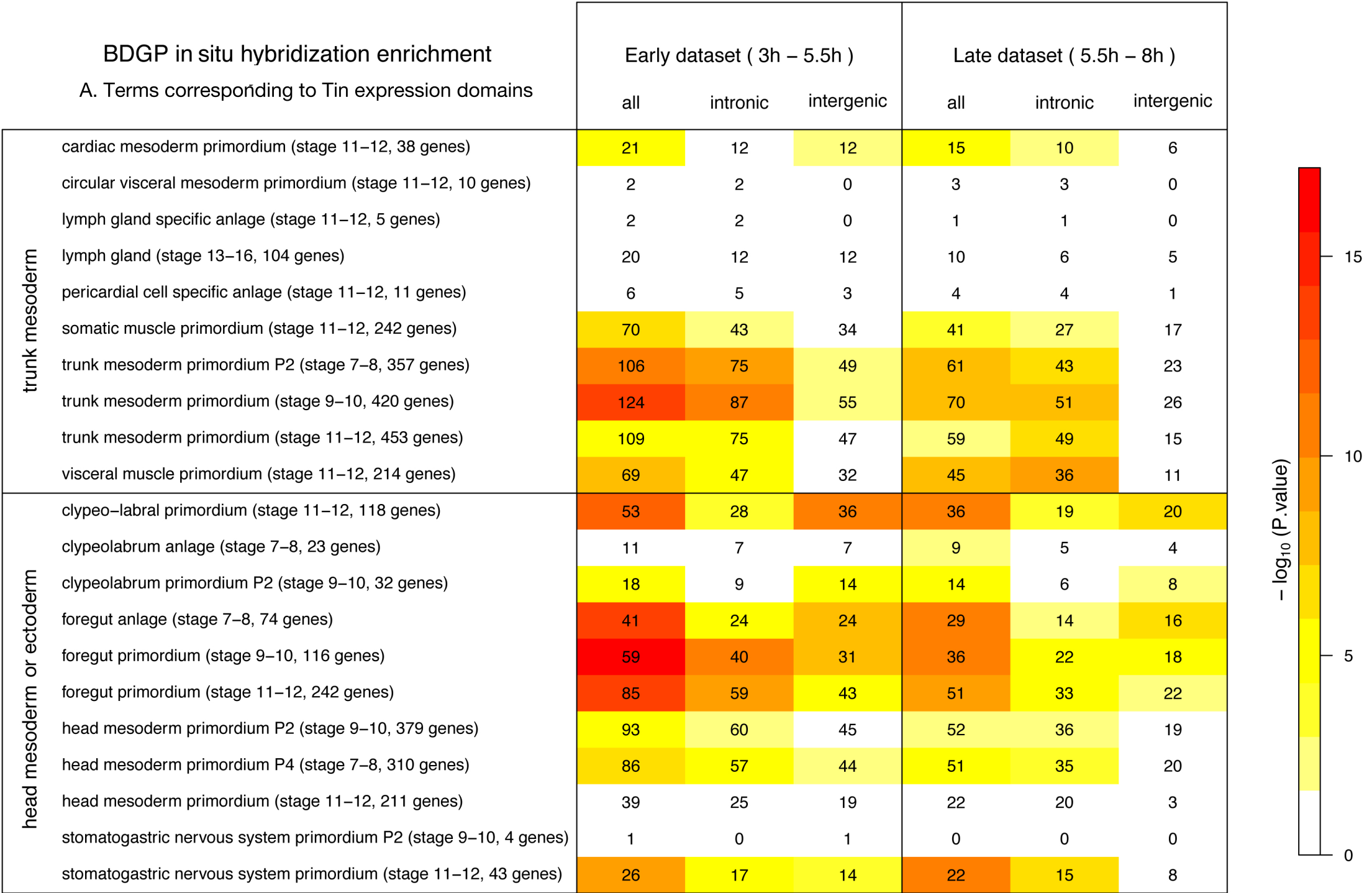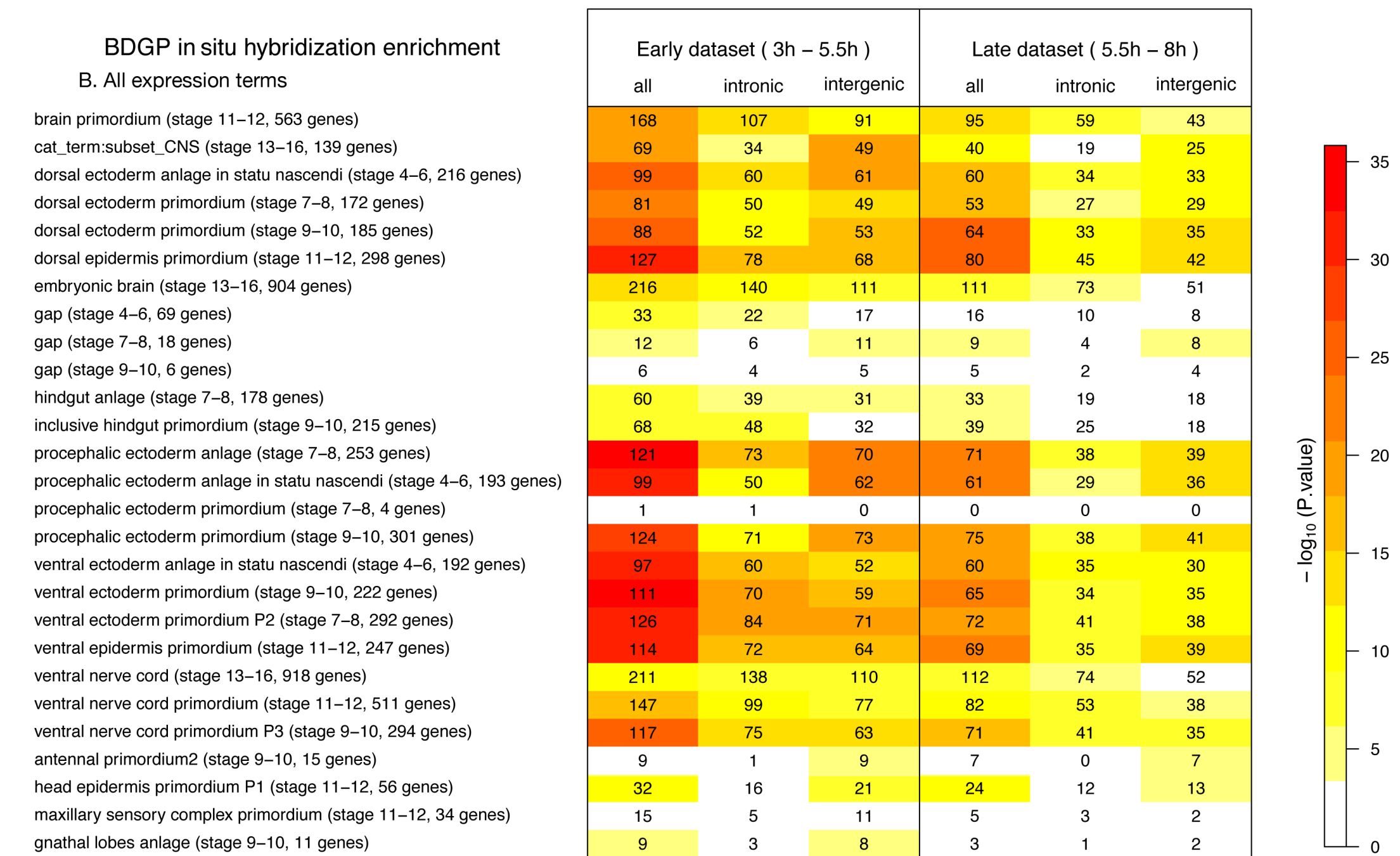

Supplement: Table S3 — Enrichment of BDGP in situ hybridization terms. A) Terms related to Tin expression domains. B) All terms. (PDF) [file pgen.1003195.s009.pdf]
